# Tyrosine kinase-independent actions of DDR2 in tumor cells and cancer-associated fibroblasts influence tumor invasion, migration and metastasis

**DOI:** 10.1242/jcs.258431

**Published:** 2021-10-13

**Authors:** Craig E. Barcus, Priscilla Y. Hwang, Vasilios Morikis, Audrey Brenot, Patrick Pence, Maria Clarke, Gregory D. Longmore

**Affiliations:** 1 ICCE Institute, Washington University, St Louis, MO 63110, USA; 2 Department of Medicine (Oncology), Washington University, St Louis, MO 63110, USA; 3 College of Engineering, Virginia Commonwealth University, Richmond, VA 23284, USA

**Keywords:** DDR2, Cancer-associated fibroblasts, Kinase independent, Metastasis, Paracrine actions

## Abstract

Both tumor cell-intrinsic signals and tumor cell-extrinsic signals from cells within the tumor microenvironment influence tumor cell dissemination and metastasis. The fibrillar collagen receptor tyrosine kinase (RTK) discoidin domain receptor 2 (DDR2) is essential for breast cancer metastasis in mouse models, and high expression of DDR2 in tumor and tumor stromal cells is strongly associated with poorer clinical outcomes. DDR2 tyrosine kinase activity has been hypothesized to be required for the metastatic activity of DDR2; however, inhibition of DDR2 tyrosine kinase activity, along with that of other RTKs, has failed to provide clinically relevant responses in metastatic patients. Here, we show that tyrosine kinase activity-independent action of DDR2 in tumor cells can support Matrigel invasion and *in vivo* metastasis. Paracrine actions of DDR2 in tumor cells and cancer-associated fibroblasts (CAFs) also support tumor invasion, migration and lung colonization *in vivo*. These data suggest that tyrosine kinase-independent functions of DDR2 could explain failures of tyrosine kinase inhibitor treatment in metastatic breast cancer patients and highlight the need for alternative therapeutic strategies that inhibit both tyrosine kinase-dependent and -independent actions of RTKs in the treatment of breast cancer.

This article has an associated First Person interview with the first author of the paper.

## Abstract (precis)

**Summary:** The collagen receptor DDR2, a receptor tyrosine kinase, exhibits tyrosine kinase-independent functions that influence paracrine regulation of breast tumor cell invasion and breast cancer metastasis.

## INTRODUCTION

In epithelial tumors, such as breast cancer, abnormal or dysregulated receptor tyrosine kinase (RTK) signaling is common. This can result from direct gain-of-function mutations to RTKs, changes in their level of expression or alterations in their downstream signaling components (Regad, 2015). In breast cancer, expression of the RTK family of epidermal growth factor receptors (EGFRs) can be a clinical marker, and treatments directly targeting the EGFR family have proven effective in these clinical subtypes. Dysregulated RTK signaling is also associated with breast cancer progression to metastasis (Butti et al., 2018).

In addition to EGFR, other RTKs play a role in breast cancer development and progression. These include fibroblast growth factor receptors (FGFRs), c-Met (also known as MET), and the fibrillar collagen discoidin domain receptor 2 (DDR2). DDR2 is of particular interest as its actions in both tumor cells and tumor stromal cells within the primary tumor environment contribute to metastasis regulation (Corsa et al., 2016). High DDR2 expression in human triple-negative breast cancer is strongly correlated with poor outcomes and metastatic disease (Toy et al., 2015). In breast tumor cells, DDR2 regulates tumor cell collective invasion by sustaining a SNAIL1 (SNAI1)-mediated epithelial–mesenchymal transition (EMT) invasive program, particularly in keratin 14 (KRT14)-positive leader cells (Zhang et al., 2013; Hwang et al., 2019), as well as full activation of collagen-binding integrins (Bayer et al., 2019). In the breast cancer stroma, the action of DDR2 in cancer associated fibroblasts (CAFs) is particularly important for force-mediated remodeling of collagen fibers in the tumor extracellular matrix (ECM), thereby controlling the mechanical properties (e.g. stiffness) of the breast tumor stroma (Zhang et al., 2016; Bayer et al., 2019). Fibroblasts within the tumor microenvironment influence tumor cell invasion and motility through direct contact, paracrine actions, and production and remodeling of the ECM (Erdogan and Webb, 2017; Houthuijzen and Jonkers, 2018; Takai et al., 2018). DDR2 action in CAFs can also increase tumor cell invasiveness through paracrine regulation (Corsa et al., 2016). These data make DDR2 an attractive therapeutic target for the prevention or treatment of metastatic breast cancer.

Stage III and IV breast cancer patients have been treated with tyrosine kinase inhibitors (TKIs), including some that inhibit DDR2 (e.g. dasatinib and imatinib), alone and in combination with other therapies (Day et al., 2008; Bai et al., 2014). Unfortunately, this treatment has minimal impact on clinical outcomes (Finn et al., 2011; Herold et al., 2011; Mitri et al., 2016; Schott et al., 2016). Potential explanations for the insufficient clinical responses could include: *de novo* or acquired resistance of RTKs to TKIs (Ohashi et al., 2013); acquisition of other contributing mutations outside of RTKs as a result of extensive pretreatment of patients in these trials (Clarke et al., 2015); the presence of a desmoplastic or fibrotic tumor stroma that reduces chemotherapeutic response rates (Schober et al., 2014); and the possible contribution of tyrosine kinase-independent actions of RTKs, which would not be impacted by treatment with TKIs (Weihua et al., 2008; Perrault et al., 2011; Tesfay et al., 2016).

We set out to determine whether DDR2 exhibited tyrosine kinase-independent actions in tumor cells and tumor stromal cells, and if so, what the impact on tumor cell biology in *ex vivo* culture systems and *in vivo* would be. We have found that in both human and mouse breast tumor cell lines and breast tumor CAFs, DDR2 does indeed exhibit tyrosine kinase-independent actions that can support tumor cell invasion and metastatic progression *in vivo*. These data suggest that tyrosine kinase-independent functions of DDR2 could explain failures of TKI treatment in metastatic breast cancer patients.

## RESULTS

### Production and characterization of cell lines

**Fig. 1.**
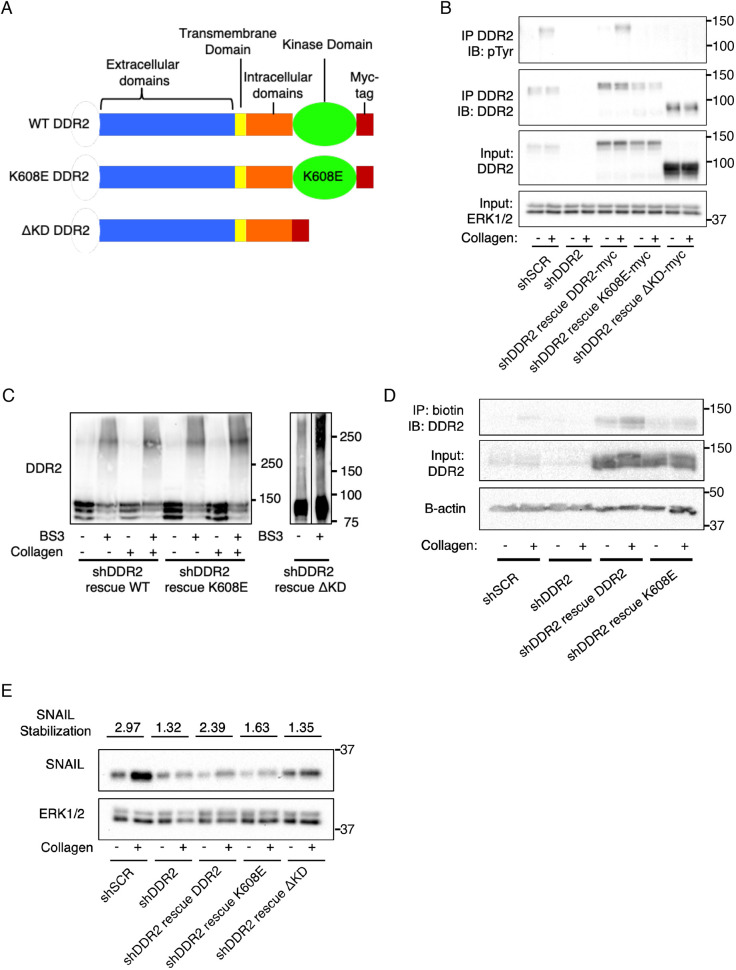
**Characterization of cell lines.** (A) Diagram showing DDR2 rescue constructs*.* (B) Western blot analyses of expression of the indicated myc-tagged DDR2 rescue constructs in BT549 cells depleted of DDR2 (shDDR2) plated on plates with or without collagen I coating for 6 h. DDR2 was immunoprecipitated (IP) using anti-DDR2 antibodies, and bound products were immunoblotted (IB) using anti-phosphotyrosine (pTyr) or anti-DDR2 antibodies. Input controls are 10% of total lysate used for IP, probed using the indicated antibodies. Anti-ERK1/2 is shown as a loading control. (C) BS^3^ crosslinking of intact DDR2-depleted BT549 cells expressing the indicated DDR2 rescue constructs, treated as in B. Lysates were western blotted for DDR2 after separation on a low percentage polyacrylamide gel. (D) Intact BT549 cells were treated as in B, then incubated with NHS-PEG-Biotin, lysed, and immunoprecipitated with streptavidin. Bound products were western blotted with the indicated antibodies (B-actin, β actin loading control). Input controls are 10% of total lysate used for IP. (E) BT549 cells were plated on plates with or without 2 mg/ml collagen I coating for 6 h before lysis. Lysates were western blotted for SNAIL1. Quantification of relative SNAIL1 level in response to collagen stimulation is listed above each lane. ERK1/2 blot is shown as a loading control. In B–E, molecular masses are indicated in kDa. Blots shown are representative of three experiments.

To determine the importance of the intracellular tyrosine kinase activity and tyrosine kinase domain of DDR2 in supporting breast tumor cell invasion, migration and metastasis, we depleted DDR2 using shRNA treatment in a set of human (BT549, Hs578 T and MDA-MB-231) and mouse (4T1) breast tumor cell lines. In DDR2-depleted BT549 and 4T1 cells, we then rescued DDR2 expression by transducing cells with the following C-terminal (cytoplasmic) myc-tagged RNAi-resistant isoforms of species-specific DDR2: wild-type DDR2 (WT), K608E (a tyrosine kinase-inactivating point mutant) and ΔKD (a cytoplasmic truncation of the tyrosine kinase domain) (Fig. 1A). The cytoplasmic tail truncation removed the entire tyrosine kinase domain while retaining the intracellular juxtamembrane domain critical for receptor dimerization and collagen binding (Kim et al., 2014). The ΔKD mutant was chosen as, although tyrosine kinase inactive, it can be used to assess whether cytoplasmic signaling adapters recruited to this domain are important for tyrosine kinase-independent actions of DDR2.

Western blots of total cell extracts from rescued cell lines revealed that expression of WT and K608E rescue isoforms were equivalent and were at levels 3–4 times greater than that of endogenous DDR2 in the parental cell line, whereas the level of ΔKD expression was much greater than that of either WT or K608E rescue expression (Fig. 1B). DDR2 tyrosine phosphorylation in response to collagen I binding, as determined by DDR2 immunoprecipitation and subsequent immunoblotting of bound products using an anti-phosphotyrosine antibody, was not detected in both K608E- and ΔKD-rescued cells but was robust in cells rescued with WT DDR2 (Fig. 1B). Similar results were observed in mouse 4T1 breast tumor cells, as well as in mouse breast tumor CAFs (Figs S1A and S1B, respectively).

DDR2 is present at the cell surface as preformed dimers in the absence of ligand, which allows for binding to fibrillar collagens and subsequent autophosphorylation of tyrosine residues in the cytoplasmic tyrosine kinase domain. Multimerization of DDR2 dimers at the cell surface in response to collagen engagement may also be important for this activity (Leitinger, 2003; Fu et al., 2013b). To determine whether WT, K608E, and ΔKD were present at the cell surface and dimerize or multimerize, we utilized the membrane impermeant, non-cleavable, water-soluble chemical crosslinker bis(sulfosuccinimidyl)suberate (BS^3^). BS^3^ crosslinking resulted in a molecular weight shift of DDR2 corresponding to dimerization of both WT and K608E in the absence and presence of collagen (Fig. 1C). In ΔKD-rescued cells, surface dimers were also detected (Fig. 1C). Cell surface expression of DDR2 rescue isoforms was also confirmed by utilizing membrane impermeant biotin labeling (Fig. 1D). Thus, all rescue isoforms of DDR2 were appropriately expressed at the cell surface in dimeric or multimeric forms.

Collagen I-induced stabilization of the EMT marker SNAIL1 is regulated by DDR2 and is dependent on DDR2 tyrosine kinase activity (Zhang et al., 2013). In WT-rescued cells, SNAIL1 was stabilized to a level similar to that observed in parental cell lines, whereas K608E- and ΔKD-rescue cell lines all exhibited reduced SNAIL1 stabilization in response to collagen I (Fig. 1E).

To determine whether there were changes in DDR2 signaling in this panel of tumor cell lines, we utilized a commercially available phosphorylation array to measure phosphorylation of downstream signaling mediators in response to collagen I. In both parental BT549 cells and WT-rescued cells there was increased phosphorylation of FGR, HSP27 (also known as HSPB1), JNK (JNK1–JNK3, also known as MAPK8–MAPK10), p38α (also known as MAPK14), PDGFR-β, PLC-γ (also known as PLCG1) and STAT2 when compared to that in DDR2-depleted cells (Fig. S1C). Compared to DDR2-depleted cells, those rescued with K608E or ΔKD also exhibited increased phosphorylation of the same proteins but not to the same extent as observed with WT-rescued cells (Fig. S1C). In both parental BT549 cells and WT-rescued cells there was decreased phosphorylation of AKT proteins (AKT1, AKT2 and AKT3) STAT3 and STAT6 when compared to that in DDR2-depleted cells (Fig. S1D). Compared to DDR2-depleted cells, those rescued with K608E or ΔKD also exhibited decreased phosphorylation of the same proteins but not to the same extent as observed with WT-rescued cells (Fig. S1D).

### Variable requirement of the DDR2 tyrosine kinase domain and DDR2 tyrosine kinase activity for breast tumor cell invasion and migration

**Fig. 2.**
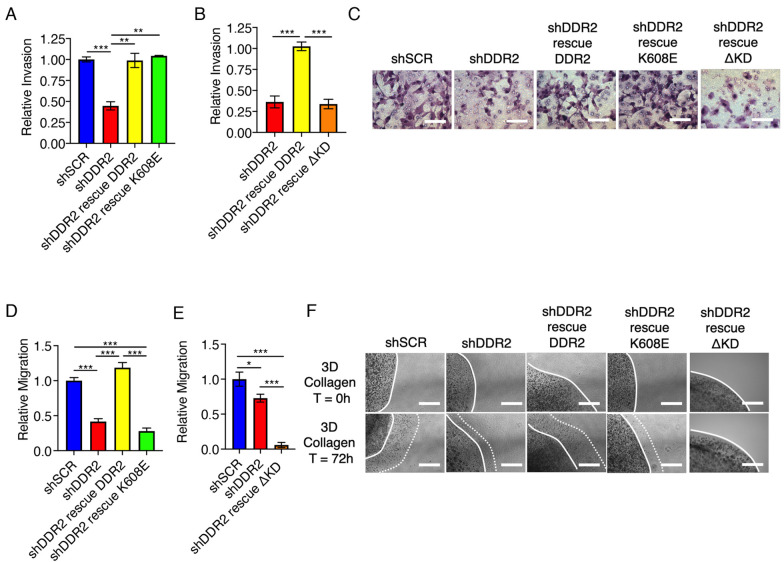
**Variable requirement of DDR2 tyrosine kinase domain or tyrosine kinase activity for breast tumor cell invasion and migration.** (A,B) Invasion of DDR2-depleted (shDDR2) BT549 cells with the indicated DDR2 rescue through Matrigel in Transwell devices at 24 h, compared to invasion by shSCR cells. (C) Representative images of Matrigel invasion for the indicated cell lines. Cells are stained using HEMA-3 stain. Scale bars: 75 μm. (D,E) Migration of DDR2-depleted BT549 cells with the indicated DDR2 rescue through 3D collagen I at 72 h compared to migration of shSCR cells. (F) Representative images of 3D collagen migration at *T*=0 h (top row) and *T*=72 h (bottom row). White line indicates cell boundary at *T*=0 h, dashed line indicates cell boundary at *T*=72 h. Data in A,B,D and E are mean±s.e.m. of three experiments. *P<0.05; **P<0.01; ***P<0.001 (one-way ANOVA with Tukey's post-hoc test). Scale bars: 50 µm.

The action of DDR2 in breast tumor cells is critical for their capacity to invade through the basement membrane and migrate through collagen-rich ECM so as to metastasize (Zhang et al., 2013). Basement membrane invasion was assessed by measuring tumor cell movement through basement membrane-derived Matrigel in Boyden chambers, whereas ECM migration was measured by embedding aggregated tumor cells in a 3D collagen I matrix. As anticipated, in all breast tumor cell lines (human BT549, Hs578 T and MDA-MB-231 cells, as well as mouse 4T1 cells) depletion of DDR2 inhibited Matrigel invasion (Fig. 2A; Fig. S2A–D) and, for BT549 and 4T1 cells, migration through 3D collagen I matrices (Fig. 2D; Fig. S2E). In both human BT549 cells (Fig. 2A–C) and mouse 4T1 breast tumor cells (Fig. S2D), K608E rescued invasion through Matrigel, but ΔKD did not. In contrast, in both human and mouse tumor cells, both ΔKD and K608E did not rescue migration through 3D collagen I (Fig. 2D–F; Fig. S2E).

### Tyrosine kinase-inactive DDR2 supports tumor cell paracrine regulation of tumor cell invasion through Matrigel

**Fig. 3.**
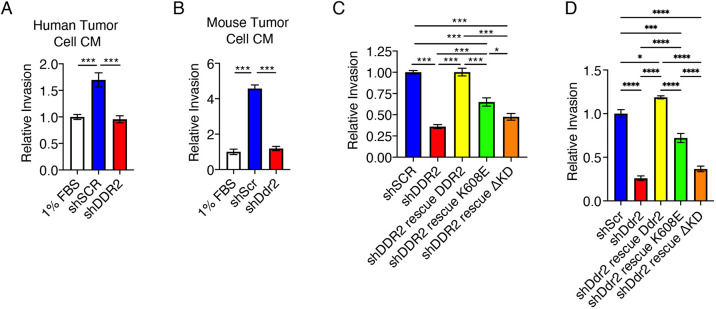
**Tyrosine kinase-inactive DDR2 supports tumor cell paracrine regulation of tumor cell invasion through Matrigel.** (A) Matrigel Transwells were treated with conditioned medium (CM) from DDR2-depleted (shDDR2) or control (shSCR) BT549 cells for 24 h, then BT549 shSCR cells were added and allowed to invade for 24 h. Invasion relative to 1% FBS (i.e. non-conditioned medium control) is shown. (B) Matrigel Transwells were treated with conditioned medium from shSCR or shDDR2 4T1 cells for 24 h, then shSCR 4T1 cells were added and allowed to invade for 48 h. Invasion relative to the 1% FBS control is shown. (C) Matrigel Transwells were treated with conditioned medium from the indicated BT549 cells for 24 h, then shSCR BT549 cells were added and allowed to invade for 24 h. Invasion relative to that on shSCR-conditioned Matrigel is shown. (D) Matrigel Transwells were treated with conditioned medium from the indicated 4T1 cells for 24 h, then shSCR 4T1 cells were added and allowed to invade for 48 h. Invasion relative to that on shSCR-conditioned Matrigel is shown. Data are mean±s.e.m. of three experiments. *P<0.05; ***P<0.001; ****P<0.0001 (one-way ANOVA with Tukey's post-hoc test).

Breast tumor cells secrete many factors that can impact tumor cell invasion in a paracrine or autocrine manner. They do so by acting directly on tumor cells or associated stromal cells, or by modifying the tumor ECM. To determine whether the action of DDR2 in tumor cells was required for these paracrine actions, we generated conditioned medium from tumor cells expressing various DDR2 receptor mutants. The conditioned medium was concentrated through a 10 kDa filter to remove peptide fragments, and then Matrigel plugs were pretreated with equivalent amounts of conditioned medium prior to the addition of parental tumor cells (i.e. cells expressing endogenous WT DDR2). The conditioned medium from both parental human BT549 and MDA-MB-231 (Fig. 3A; Fig. S3), and mouse 4T1 (Fig. 3B) breast tumor cells enhanced invasion of WT breast tumor cells through Matrigel, and this required the presence of DDR2, as this activity was absent when conditioned medium from DDR2-depleted tumor cells was used. Re-expression of WT DDR2 in DDR2-depleted tumor cells completely rescued this activity (Fig. 3C,D).

Conditioned medium from both human and mouse tumor cells rescued with K608E also enhanced tumor cell invasion through Matrigel, but only partially when compared to the effect of conditioned medium from WT DDR2-expressing tumor cells (Fig. 3C,D). Conditioned medium from both human and mouse tumor cells rescued with ΔKD failed entirely to enhance invasion through Matrigel (Fig. 3C,D).

These results indicate that tyrosine kinase-inactive DDR2 (K608E) was capable of supporting tumor cell paracrine regulation of Matrigel invasion by tumor cells. In contrast, DDR2 lacking the tyrosine kinase domain (ΔKD) was not.

### Tyrosine kinase-inactive DDR2 partially rescues lung metastasis of tumor cells *in vivo*

**Fig. 4.**
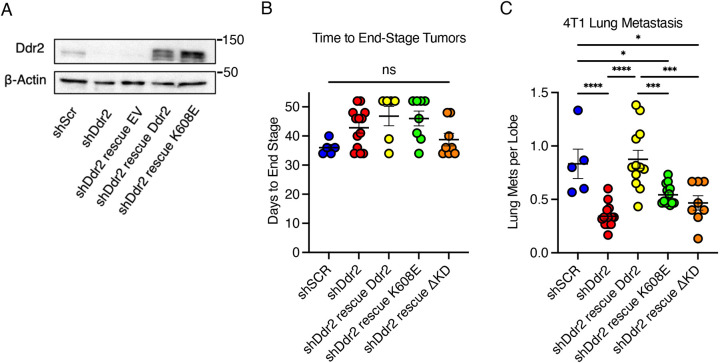
**Tyrosine kinase-inactive DDR2 partially rescues lung metastasis of tumor cells *in vivo*.** (A) Lysates from the indicated 4T1 cell lines (shDdr2, DDR2 depleted; rescue EV, empty vector control) were immunoblotted using the indicated antibodies. β-actin is shown as a loading control. Molecular masses are shown in kDa. Blots are representative of three experiments. (B) Time to end-stage tumors (2 cm diameter) for the indicated orthotopically transplanted 4T1 tumor cells. Each symbol represents an individual mouse. Mean and s.e.m. are indicated. (C) Lung metastases of mice orthotopically transplanted with the indicated 4T1 tumor cells. Data are represented as lung metastases (Lung Mets) per lobe, averaged over three step-sections of at least 50 µm. Metastases were only counted if more than ten tumor cells were present. Each symbol represents an individual mouse. Mean and s.e.m. are indicated. A minimum of 5 animals (shSCR) and a maximum of 16 animals (shDdr2) were used. *P<0.05; ***P<0.001; ****P<0.0001; ns, not significant (one-way ANOVA with Tukey's post-hoc test).

The action of DDR2 in breast tumor cells is critical for *in vivo* lung metastasis in syngeneic orthotopic transplant and spontaneous genetically engineered mouse cancer models (Corsa et al., 2016; Grither and Longmore, 2018). To determine whether the partial effects of tyrosine kinase-inactive DDR2 in tumor cells was relevant *in vivo*, we generated Balb/c-derived 4T1 mouse mammary tumor cell lines depleted of DDR2 (using shRNA) and lines rescued with species-specific WT, K608E or ΔKD DDR2 isoforms. Rescue isoforms were expressed at levels greater than that of endogenous DDR2 (Fig. 4A; Fig. S1A). Tumor cells were orthotopically transplanted into the inguinal mammary gland of syngeneic Balb/c females and allowed to progress to end stage (2 cm primary tumor). No significant difference in time to end-stage tumor development was observed between the various tumor cells (Fig. 4B). As previously reported, DDR2-depleted tumor cells had significantly reduced number of pulmonary metastases compared to that observed for control parental cells (expressing scrambled shRNA, shSCR) (Fig. 4C; *P*<0.001). WT-rescued cells rescued pulmonary metastasis to parental control levels (Fig. 4C). K608E-rescued cells showed reduced pulmonary metastasis compared to that of parental control tumors (*P*<0.05), but increased pulmonary metastasis compared to that of DDR2-depleted tumor cells (*P*<0.05) (Fig. 4C). ΔKD-rescued cells also exhibited a reduced number of lung metastases, equivalent to that observed for DDR2-depleted cells (Fig. 4C). Thus, breast tumor cells expressing tyrosine kinase-inactive K608E.DDR2 exhibited real but diminished lung metastases *in vivo*. In other words, tumor cell tyrosine kinase-inactive DDR2 supported breast cancer metastasis *in vivo*.

### DDR2-regulated CAF paracrine regulation of tumor cell invasion through Matrigel

**Fig. 5.**
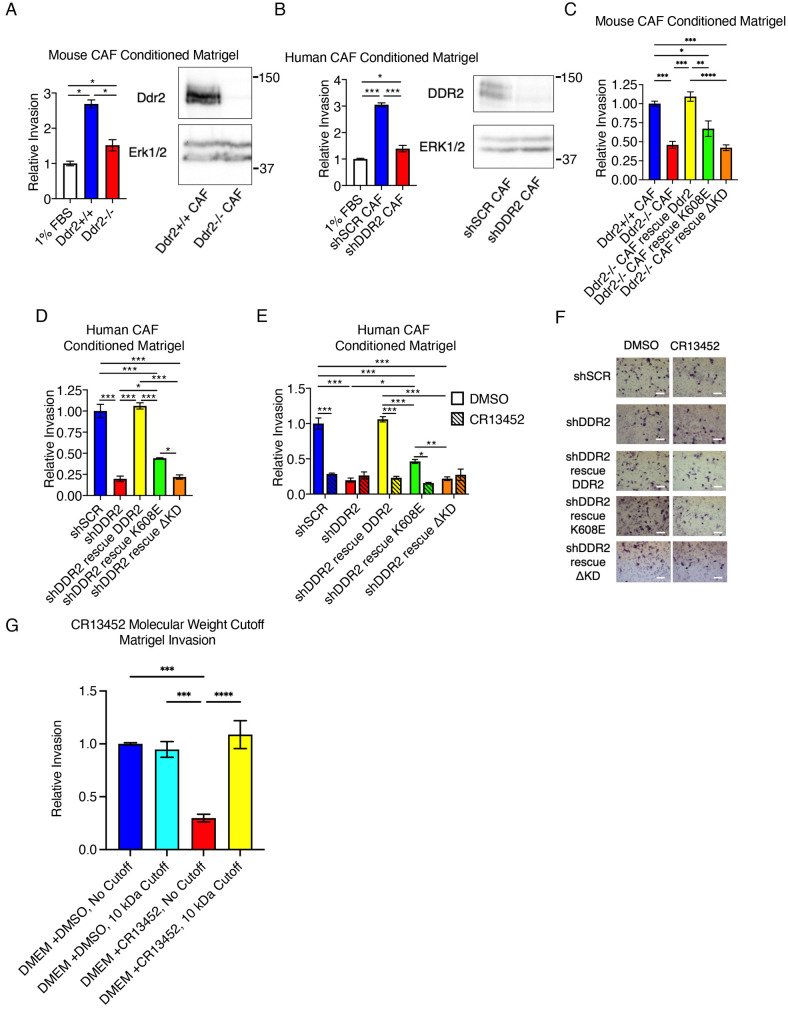
**DDR2-regulated CAF paracrine regulation of tumor cell invasion through Matrigel**. (A) Matrigel Transwells were treated with conditioned medium from mouse CAFs of the indicated genotype for 24 h, then shSCR 4T1 cells were added and allowed to invade for 48 h. Left, invasion is shown relative to the 1% FBS-treated control. Right, western blot for DDR2 expression in indicated mouse CAFs. ERK1/2 is shown as a loading control. (B) Matrigel Transwells were treated with conditioned medium from the indicated human CAF cell lines (shDDR2, DDR2-depleted) for 24 h, then shSCR BT549 cells were added and allowed to invade for 24 h. Left, invasion is shown relative to the 1% FBS-treated control. Right, western blot for DDR2 expression in indicated human CAFs. ERK1/2 is shown as a loading control. (C) Matrigel Transwells were treated with conditioned medium from mouse CAFs of the indicated genotype for 24 h, then shSCR 4T1 cells were added and allowed to invade for 48 h. Invasion is shown relative to that on Matrigel treated with *Ddr2*^+/+^ CAF-conditioned medium. (D) Matrigel Transwells were treated with conditioned medium from the indicated human CAF cell lines for 24 h, then shSCR BT549 cells were added and allowed to invade for 24 h. Invasion is shown relative to that on Matrigel treated with human shSCR CAF-conditioned medium. (E) Matrigel transwells were treated with conditioned medium from the indicated human CAF cell lines treated with either the DDR2 allosteric inhibitor CR13452 or DMSO for 24 h, then shSCR BT549 cells were added and allowed to invade for 24 h. Invasion relative to that on Matrigel treated with conditioned medium from DMSO-treated control shSCR BT549 cells is shown. (F) Representative images of the experiment in E. Cells are stained using HEMA-3 stain. Scale bar: 75 μm. (G) The small-molecule DDR2 allosteric inhibitor CR13452 is removed from conditioned media by molecular weight cut-off concentration. DMEM was added to tissue culture plastic in the absence of cells with either 10 µM CR13452 or DMSO for 24 h at 37C and 5% CO_2_ to mimic experiments in the presence of cells. After 24 h, a portion of the medium was collected and concentrated through a 10 kDa molecular weight cut-off concentrator. Matrigel transwells were treated with equivalent volumes and allowed to condition for 24 h, then WT BT549 cells were added and allowed to invade for 24 h. For a positive control of CR13452 activity, CR13452 was added directly with cells in one triplicate experiment with DMEM (red bar). Data in A–E,G are mean±s.e.m. of three experiments. *P<0.05; **P<0.01; ***P<0.001; ****P<0.0001 (two-way ANOVA with Tukey post-hoc test).

In addition to its tumor cell-intrinsic actions, the action of DDR2 in breast tumor stromal CAFs also contributes to lung metastases (Corsa et al., 2016; Bayer et al., 2019)*.* Thus, we asked whether DDR2 signaling in CAFs also affected paracrine regulation of tumor cell invasion and migration. To do so, we utilized CAFs derived from *Ddr2*^+/+^ and ubiquitous *Ddr2*^−/−^ (genetic *Ddr2*-null cells) MMTV-PyMT mouse tumors (Fig. 5A). *Ddr2*^−/−^ CAFs were rescued by re-expression of species-specific WT, K608E or ΔKD DDR2 isoforms. We also generated human CAFs depleted of DDR2 (using shRNA; Fig. 5B) and rescued these with RNAi-resistant species-specific WT, K608E or ΔKD isoforms.

Conditioned medium from both human and mouse parental CAFs increased WT tumor cell invasion through Matrigel, and this activity required the presence of DDR2, as conditioned medium from DDR2-depleted CAFs did not enhance tumor cell invasion (Fig. 5A,B). Conditioned medium from WT-rescued mouse and human DDR2-depleted CAFs completely rescued this activity (Fig. 5C,D). Conditioned medium from DDR2-depleted CAFs rescued with K608E partially rescued Matrigel invasion, whereas conditioned medium from DDR2-depleted CAFs rescued with ΔKD did not (equivalent to DDR2-depleted conditioned medium) (Fig. 5C,D).

In another approach to confirm that DDR2 activity in CAFs is important for paracrine regulation of tumor cell invasion through Matrigel, we utilized a small-molecule allosteric inhibitor of DDR2 that interacts with the extracellular domain of DDR2 (CR13452 Rottapharm Biotech S.r.l., Monza, Italy). All CAF cell lines were treated with CR13452, or not, and conditioned medium was harvested, concentrated through a 10 kDa filter to remove any residual CR13452 and then used to pretreat Matrigel. Following treatment of CAFs with CR13452, conditioned medium from all previously active DDR2-expressing CAFs, including K608E-expressing cells, no longer enhanced tumor cell invasion (Fig. 5E,F). The effect was equivalent to that of conditioned medium from DDR2-depleted CAF control experiments. The inhibitory action of CR13452 was specific for DDR2 (i.e. was not an off-target effect) as treatment of CAFs depleted of DDR2 had no further effect (Fig. 5E,F). To control for possible inhibitory effects of any residual CR13452 on added WT tumor cells used in the assay, tissue culture medium with or without CR13452 was concentrated through a 10 kDa molecular weight cut-off concentrator, or not, before adding to Matrigel. Filtration completely removed all inhibitory activity attributed to CR13452 (Fig. 5G). In summary, as observed in breast tumor cells, tyrosine kinase-inactive DDR2 (K608E) signaling in CAFs supported paracrine regulation of Matrigel invasion by tumor cells; however, the tyrosine kinase domain of DDR2 (ΔKD) was required.

### DDR2-regulated CAF paracrine regulation of tumor cell migration through 3D collagen I

**Fig. 6.**
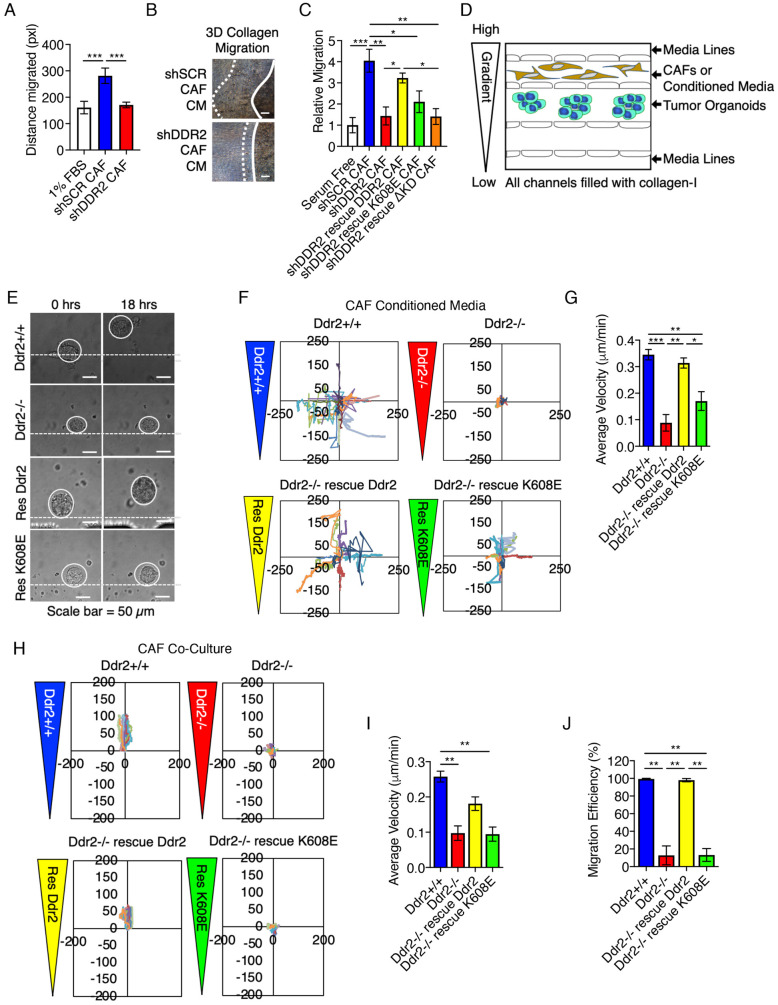
**DDR2-regulated CAF paracrine regulation of tumor cell invasion through collagen I.** (A) 3D collagen I plugs containing shSCR BT549 cells were treated with conditioned medium (CM) from shSCR or DDR2-depleted (shDDR2) human CAFs. Invasion was allowed to proceed for 24 h in the presence of CAF-conditioned medium and is shown relative to that in 1% FBS-treated 3D collagen I (pxl, pixel). Data are mean±s.e.m. of three experiments. (B) Representative images of the experiment in A. White line indicates cell boundary at *T*=0 h, dashed line indicates cell boundary at *T*=72 h. Scale bar: 50 μm. (C) 3D collagen I assays, as in A, treated with conditioned medium from the indicated human CAF cell lines. Invasion is shown relative to that in 1% FBS-treated 3D collagen I. Data are mean±s.e.m. of three experiments. (D) Model representation of the microfluidic device used to measure primary MMTV-PyMT breast tumor organoid directed collective migration through 3D collagen I. Freshly prepared breast tumor organoids are placed in the central chamber within 3D collagen I. All microfluidic experiments were performed in the presence of <2% oxygen. (E) Representative images of WT organoid collective migration through 3D collagen I treated with conditioned medium from the indicated CAFs (Res, rescue). Left panel, *T*=0 h; right panel, *T*=18 h. Dashed lines show tumor organoid location at *T*=0, circles indicate tumor organoid boundary. (F) Conditioned medium from the indicated mouse CAFs was added to the upper medium source input to generate a gradient (see Materials and Methods). Organoid migration over 16 h was tracked and plotted in the *X* and *Y* planes of the gradient (in µm). (G) Average velocity (µm/min) of tumor organoid movement for the experiment shown in E. A minimum of three organoids from three different mice were tracked. Data are mean±s.e.m. (H) Mouse CAFs of the indicated genotype were added to the proximal chamber of the microfluidic device, and movement of tumor organoids in the central chamber was analyzed as in E. Each experiment was performed twice with different tumor organoid preparations. Only devices in which CAFs had not migrated into the center chamber were analyzed. Distances are plotted in µm. (I) Average velocity (µm/min) of tumor organoids in the experiment shown in G. (J) Migration efficiency of tumor organoid collective migration in the experiment shown in G, normalized to the *Ddr2*^+/+^ CAF condition. Data in I and J are mean±s.e.m. of a minimum of three organoids. *P<0.05; **P<0.01; ***P<0.001 (one-way ANOVA with Tukey's post-hoc test).

We next evaluated the role of the paracrine activity of DDR2 in CAFs in supporting migration of tumor cells through 3D collagen I gels. Collagen I matrices pretreated with CAF-conditioned medium resulted in significantly increased tumor cell migration through 3D collagen I, whereas pretreatment with conditioned medium from DDR2-depleted CAFs did not (Fig. 6A,B). Conditioning 3D collagen matrices with medium from WT-rescued CAFs mimicked parental controls (Fig. 6C). Conditioned medium from K608E-rescued CAFs rescued, partially, tumor cell migration in collagen I gels, whereas medium from ΔKD-rescued CAFs failed to support migration through 3D collagen I (equivalent to DDR2-depleted CAFs) (Fig. 6C).

*In vivo* invading and migrating breast tumor cells are exposed to hypoxia and chemokine or cytokine signaling gradients (not uniform concentrations). Moreover, they often invade collectively and not individually. Therefore, we made use of a previously described microfluidic device (Fig. 6D) (Hwang et al., 2019) to measure collective migration of primary mouse MMTV-PyMT breast tumor organoids through 3D collagen I matrices under hypoxic conditions and in response to CAF-conditioned medium gradients. PyMT mouse breast tumor organoids were placed in the central channel in 3D collagen I (<2% O_2_) and then subjected to a mouse CAF-conditioned medium gradient (ranging from full concentration to no medium). Conditioned medium from parental *Ddr2*^+/+^ CAFs supported collective migration of breast tumor organoids, whereas medium from *Ddr2*^−/−^ CAFs did not (Fig. 6E and F, quantified in G). Conditioned medium from WT-rescued *Ddr2*^−/−^ CAFs completely rescued the deficit of *Ddr2*^−/−^ CAF conditioned medium (Fig. 6E and F, quantified in G). Conditioned medium from K608E-rescued *Ddr2*^−/−^ CAFs also enhanced collective migration velocity compared to the effect of medium from *Ddr2*^−/−^ CAFs, but not to the same extent as the effect of conditioned medium from WT DDR2-rescued CAFs (Fig. 6E and F, quantified in G). It should be noted that there was no directionality to the collective migration of tumor organoids in the CAF-conditioned medium gradients.

To determine whether active secretion by CAFs would induce directed collective tumor organoid migration, we utilized the microfluidic device described above but added CAFs expressing various DDR2 isoforms in the channel above the tumor organoids, which were in the central channel. All cells were embedded in 3D collagen I. *Ddr2*^+/+^ and WT-rescued *Ddr2*^−/−^ CAFs enhanced both the average velocity of collective migration and directed collective migration (migration efficiency towards the upper channel containing CAFs) of tumor organoids (Fig. 6H–J). *Ddr2*^−/−^ and K608E-rescued *Ddr2*^−/−^ CAFs did not enhance either average velocity or migration efficiency in this experimental setup (Fig. 6H–J).

To control for the contribution of CAF-mediated collagen fiber remodeling to these outcomes, devices in which the CAFs migrated into the middle chamber, where tumor organoids were present, were not analyzed. We also acquired second-harmonic images (SHGs) of collagen in the upper channels (containing CAFs) and in the center of the middle channel (where organoids were). CAFs remodeled collagen fibers in the upper channel (Fig. S4A), as anticipated (Fig. S4B). SHG images of collagen organization in the middle of the central channel, where tumor organoids were present, were analyzed for fiber width, length, angle and straightness using CT-FIRE and CurveAlign software (Bredfeldt et al., 2014). We did not observe any significant change in collagen fiber organization in the middle of the central channel in any of the experimental devices (Fig. S4B–F).

In conclusion, the presence of CAFs in the device, as opposed to CAF culture medium, imparted directionality to the tumor organoid collective migration. Both situations increased the average velocity of collective migration. Taken together, these CAF paracrine experiments indicated that DDR2 signaling in CAFs was critical for CAF paracrine regulation of single and collective tumor cell migration through 3D collagen I matrices. Although tyrosine kinase-inactive DDR2 (K608E) was able to support single-cell and collective migration though collagen, it did not support directional collective migration in collagen. In contrast, the tyrosine kinase domain of DDR2 was required for all analyzed CAF paracrine actions.

### Tyrosine kinase-independent DDR2 signaling in CAFs supports tumor lung colonization *in vivo*

**Fig. 7.**
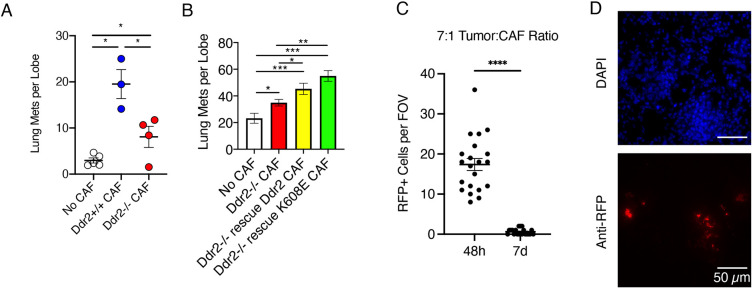
**Tyrosine kinase-independent DDR2 signaling in CAFs supports tumor lung colonization *in vivo*.** (A) Isolated syngeneic (FVB/N) PyMT tumor cells (7.5×10^5^) were co-injected with CAFs (FVB/N) of indicated genotypes (1×10^5^) in the tail vein of FVB/N female mice. After 7 days, lungs were harvested, and metastases (Mets) were counted in lung slices stained with hemotoxylin and eosin, as described in Materials and Methods. For each experimental condition, four mice were analyzed. The mean±s.e.m. is indicated. (B) PyMT tumor cells (7.5×10^5^) were co-injected with CAFs of the indicated genotypes (1×10^5^), and lung slices were analyzed as described in A. For each condition, five mice were analyzed. Data are mean±s.e.m. *P<0.05; **P<0.01; ***P<0.001 (one-way ANOVA with Tukey's post-hoc test). (C) To determine whether CAFs were present in lung colonization sites after co-injection, we generated RFP-positive (RFP+) WT mouse CAFs and co-injected them with Bo1 FVB/N mouse mammary tumor cells at the same 7:1 ratio as used in A and B, using 7×10^5^ tumor cells. After 48 h or 7 d, lungs were collected and processed as described in the Materials and Methods. Immunofluorescence was performed on formalin-fixed paraffin-embedded lung sections using anti-RFP antibodies. The total number of RFP+ cells was counted per field of view (FOV) obtained using a 10× objective. *n*=8 FOVs. The mean±s.e.m. is indicated. ****P<0.0001 (unpaired *t*-test). (D) Representative FOV of a 48-h sample from the experiment in C, obtained using a 60× objective. DAPI staining (blue) and anti-RFP immunofluorescence (red) are shown.

To determine whether the actions of DDR2 in CAFs influenced tumor cell metastatic properties *in vivo*, we co-injected mouse CAFs of *Ddr2*^+/+^ or *Ddr2*^−/−^ genotype alongside WT primary PyMT tumor cells (*Ddr2*^+/+^) into the tail vein of syngeneic FVB/N female mice and measured lung colonization after 7 days. In the presence of *Ddr2*^+/+^ CAFs, there was increased lung colonization compared to that in animals injected with tumor cells alone (Fig. 7A). Addition of *Ddr2*^−/−^ CAFs also increased lung colonization but significantly less than the addition of *Ddr2*^+/+^ CAFs (Fig. 7A). When *Ddr2*^−/−^ CAFs rescued with WT or K608E were added, the number of lung tumors per lobe was significantly increased compared to that of mice in which *Ddr2*^−/−^ CAFs were added (Fig. 7B). To ensure that injected CAFs survived in the lungs, we co-injected WT CAFs stably expressing RFP alongside the tumor cells and quantified CAF presence in lungs using immunofluorescence. At day 2 after injection, ∼20 CAFs per field of view were detected; however, by day 7 only 0–3 CAFs per field of view were present (Fig. 7C,D). These results suggested that the tyrosine kinase-independent action of DDR2 in CAFs is important for the *in vivo* regulation of tumor cell lung colonization.

## DISCUSSION

Despite the success of TKIs in the treatment of advanced lung cancer (Paez et al., 2004; Sequist et al., 2008; 2011; Marcar et al., 2019), to date, TKI treatment of late-stage metastatic breast cancer patients has failed to generate as favorable outcomes (reviewed in Nakai et al., 2016; Dickler et al., 2009). In breast cancer, there can be increased ECM deposition and remodeling that leads to changes in tumor stiffness or other physical properties, which are factors that are hypothesized to contribute to TKI failure (Senthebane et al., 2017; Wang et al., 2019). The action of the RTK DDR2 in breast tumor stromal CAFs has been shown to contribute to the development of breast tumor stiffness by influencing ECM production and mechanical remodeling through control of full activation of collagen binding integrins (Bayer et al., 2019). Here, we demonstrate that the action of DDR2 in both breast tumor cells and breast tumor stromal CAFs also affects their paracrine regulation of tumor cell invasion and migration. Importantly, tyrosine kinase-inactive DDR2 supports these effects, partially, in cell lines *ex vivo* and *in vivo*. These results suggest a role for the tyrosine kinase-independent actions of DDR2 in regulating breast cancer metastasis and could explain, in part, the ineffective responses to TKI therapies in aggressive late-stage breast cancer patients.

Tyrosine kinase-independent actions of other RTKs have been described previously (reviewed in Rauch et al., 2011; Thomas and Weihua, 2019). Activation of mitogenic signals (most notably ERK1 and ERK2, also known as MAPK3 and MAPK1) by tyrosine kinase-inactive EGFR was described over 25 years ago (Campos-González and Glenney, 1992; Selva et al., 1993; Eldredge et al., 1994). More recently, tyrosine kinase-inactive EGFR has been shown to impact not only mitogenesis (Ewald et al., 2001), but also cell survival (Weihua et al., 2008; Tan et al., 2015) and oncogenic transformation (Cho et al., 2018). Whereas a reduction of EGFR protein level can lead to cell death in cancer cells, inhibition of tyrosine kinase activity via TKI treatment does not, suggesting the presence of tyrosine kinase-independent actions of EGFR (Katreddy et al., 2018). Tumor cell and patient responses can become resistant to TKI therapies targeting EGFR (reviewed in Nakai et al., 2016). Although most of these result from acquisition of mutations in the TKI-binding sites of receptors, other studies have observed resistance due to tyrosine kinase-inactive EGFR dimerization through palmitoylation (Thomas et al., 2019). Another RTK, c-Met, also exhibits tyrosine kinase-independent activity (Tesfay et al., 2016). c-Met maintains α3β1 integrin expression and, thus, cell survival. This activity requires the extracellular and transmembrane regions of c-Met, but not the intracellular kinase domain. Rather, c-Met dimerization forms a scaffolding complex with Fas and α3β1 integrin to maintain cell adhesion and prevent apoptosis (Tesfay et al., 2016).

RTKs typically bind extracellular growth factors or cytokines, which in turn induce intracellular tyrosine autophosphorylation of the receptor itself and subsequent downstream phosphorylation of signaling targets. DDR2 (and its family member DDR1) are unique among RTKs in that they do not bind soluble ligands and instead bind to and are activated by insoluble extracellular fibrillar collagens. Previous *in vivo* data (Zhang et al., 2013; Corsa et al., 2016) and data presented herein indicate that, at least in breast cancer cell lines and breast tumors, DDR2 inhibition has little effect on cell proliferation or survival. Rather, the actions of DDR2 in tumor cells predominantly affect tumor cell motility and invasion. In tumor stromal cells (such as CAFs), DDR2 remodels the tumor stromal ECM into a more invasion-permissive environment (Bayer et al., 2019). Many of the tyrosine kinase-independent actions of other RTKs described above involve receptor internalization, scaffolding or dimerization, but DDR2, including tyrosine kinase-inactive DDR2, is present on the cell surface as a preformed dimer in the absence of ligand (Leitinger, 2003). Whereas DDR1 has been shown to undergo extracellular shedding (Fu et al., 2013a) and endocytosis (Fu et al., 2013b), evidence supporting DDR2 extracellular shedding or internalization and vesicular trafficking is absent.

In 3D collagen I matrices, tumor cells containing tyrosine kinase domain-truncated DDR2 (ΔKD), as opposed to tyrosine kinase-inactive DDR2 (K608E), were more inhibited in their migratory capacity (Fig. 2D,E). It is possible that cells expressing ΔKD DDR2 interact more avidly with collagen in part because ΔKD DDR2 cannot be endocytosed and cleared from the cell surface; however, we cannot exclude that this could also be a result of the significant overexpression of ΔKD DDR2 compared to that of K608E DDR2. Alternatively, or additionally, ΔKD DDR2 could block intracellular signaling of residual WT DDR2 or other receptors in a dominant inhibitory manner. This is a possibility in cells depleted of DDR2 using shRNA, as this approach does not achieve 100% depletion. However, in our CAF experiments this is unlikely, as the cells used in these experiments were genetic nulls that do not express any DDR2, and we did not observe any dominant inhibitory action (i.e. activity greater than *Ddr2*^−/−^) (see Fig. 5C and Fig. 6C).

The DDR2 tyrosine kinase domain is important for collagen fibrillogenesis and remodeling (Blissett et al., 2009). Activation of DDR2 via collagen binding can result in Src-mediated phosphorylation of three key tyrosine residues in the DDR2 kinase domain: Y736, Y740 and Y741 (Yang et al., 2005). Phosphorylation of these residues stimulates DDR2 kinase activity and the assembly of SHC and SHP signaling complexes (Iwai et al., 2013), with subsequent activation of ERK2 and stabilization of SNAIL1 in the nucleus (Tran et al., 2014). The tyrosine kinase domain of DDR2 could also serve as a scaffold domain for signaling intermediates. Within this region there are multiple serine, threonine and tyrosine residues that have the potential to be phosphorylated. Serine/threonine phosphorylation of the DDR receptors has not yet been explored and could explain the partial functioning of tyrosine kinase-inactive DDR2 receptors. If so, removal of potential serine/threonine phosphorylation sites in this domain in the ΔKD mutant could explain the more severe defects observed. Considering that fibroblasts, including CAFs, are embedded within the ECM and thus are surrounded by excess collagen I, tyrosine kinase-independent effects in CAFs may be minimal and transient. In contrast, tumor cells encounter fibrillar collagens only after invasion through the basement membrane; therefore, tyrosine kinase-independent effects of DDR2 may be more relevant in tumor cells.

The paracrine action of WT DDR2 and K608E.DDR2 in CAFs supported tumor cell invasion through Matrigel and migration (both single-cell and collective). This is perhaps surprising, because Matrigel, a basement membrane-extracted matrix, does not contain fibrillar collagens, the ligands for DDR2. However, DDR2 signaling controls the production of matrix metalloproteinases and specifically can enhance the activity of MT1-MMP (also known as MMP14) (Majkowska et al., 2017). Perhaps it is this activity that facilitates Matrigel invasion by parental tumor cells.

In summary, we believe we have shown that tyrosine kinase-independent actions of DDR2 in breast tumor cells and breast tumor CAFs contribute to metastatic regulation of breast cancer, in particular through regulation of the secretome (i.e. paracrine regulation). As such, these data suggest that targeting tyrosine kinase activity alone (for example, using TKIs) may not be sufficient treatment to prevent metastatic disease, and this possibly explains the poor clinical responses to TKI treatment observed to date. Pharmacologic targeting of DDR2 with an allosteric inhibitor such as CR13452, which acts via the extracellular region, blocks all DDR2-dependent cellular effects, including tyrosine kinase-independent effects, in tumor cells and CAFs. Thus, adjuvant therapy directed at RTKs needs to consider not only targeting both tumor cells and tumor stromal cells (including CAFs), but also the development of agents that inhibit both tyrosine kinase-dependent and tyrosine kinase-independent action.

## MATERIALS AND METHODS

### Reagents

Antibodies used for these studies were as follows: anti-DDR2 (#12133, Cell Signaling Technology), anti-ERK1 and ERK2 (ERK1/2; #4695, Cell Signaling Technology), anti-myc-tag clone 9B11 (#2276, Cell Signaling Technology), anti-SNAIL (Cell Signaling Technology, #3879) anti-phosphotyrosine clone 4G10 (#05-231, Millipore), anti-phospho-DDR2 Y720 (MAB25382, R&D Systems), anti-vinculin (V9131, Sigma-Aldrich), anti-β-actin (A1978, Sigma-Aldrich). The kinome array was purchased from R&D Systems and analysis performed according to the manufacturer's recommendation (R&D Systems; ARY003C). Phalloidin–Alexa Fluor 488 (#A12379) was purchased from Thermo Fisher Scientific. Growth factor reduced (GFR) Matrigel was purchased from BD Biosciences (#354230). High-density rat tail collagen I was purchased from Fisher Scientific (#CB354249). Transwell inserts (8 µm pore size) were purchased from Sigma-Aldrich (#cls3422). 3 kDa and 10 kDa protein cut-off concentrators were purchased from Thermo Fisher Scientific (#88517). Novel small-molecule CR13452 was provided by Rottapharm Biotech S.r.l.

### Cell lines and lentiviral constructs

All cell lines were initially purchased from ATCC and maintained in mycoplasma-free culture. Human cancer associated fibroblasts (CAFs) were a gift from Dr McAllister (Harvard Medical School, Boston, MA). Mouse CAFs were derived from MMTV-PyMT FVB/N tumors of either *Ddr2*^+/+^ or *Ddr2*^−/−^ animals, as previously described (Bayer et al., 2019). All cells were cultured in DMEM (Gibco) supplemented with 10% heat-inactivated FBS (Sigma-Aldrich) and penicillin and streptomycin (Gibco) at 37°C and 5% CO_2_. shRNA and expression constructs were packaged into pFLRu (Feng et al., 2010) or pLVX (NovoPro) lentiviral vectors containing either puromycin or hygromycin mammalian selection cassettes. Human and mouse DDR2 shRNA constructs have been previously derived (Zhang et al., 2013; Grither et al., 2018). Rescue constructs of C-terminal myc-tagged RNAi-resistant species-specific DDR2 wild-type (WT), kinase inactive (K608E), or kinase domain truncation (ΔKD) were cloned into pLVX viral vectors containing a hygromycin selection cassette. For the full-length isoform (WT and K608E), myc was fused to amino acid E855; for ΔKD, myc was fused to amino acid G517. Virus was produced in HEK293T packing cells, cell lines were infected and pool selected, and then clonal cell lines were derived from surviving pools. ΔKD was generated by truncating DDR2 after S539, which removed the kinase domain entirely while retaining the intracellular juxtamembrane domains critical for dimerization (Kim et al., 2014). Cell lines were selected with 3 µg/ml puromycin or 800 µg/ml hygromycin (BT549) or 100 µg/ml hygromycin (CAF lines) and kept under selection during propagation.

### Immunoblotting and immunoprecipitation

Immunoblotting was performed as previously described (Bayer et al., 2019). Briefly, cells were lysed in RIPA lysis buffer including 0.5% SDS, the protein concentration was quantified, and samples were subjected to SDS–PAGE then transferred to PVDF membranes and probed using the following antibody dilutions: DDR2 (1:1000), ERK1/2 (1:5000), phospho-DDR2 Y720 (1:1000), SNAIL (1:1000), β-actin (1:15,000), myc tag (1:1000), phosphotyrosine (1:1000). Immunoprecipitation was performed as previously described (Grither and Longmore, 2018). Briefly, for non-denatured immunoprecipitations, cells were harvested in RIPA buffer without SDS, the protein concentration was quantified and samples were precleared for 1 h in protein A/G–agarose beads (GE Healthcare; 17-0618-01) and incubated with either anti-DDR2 (1:100) or anti-myc-tag (1:100) antibodies overnight at 4°C. Protein–antibody complexes were isolated using protein A/G agarose beads and washed extensively in lysis buffer, and immunoblotting was performed as described above.

### Cell surface labeling and crosslinking

Cells were plated on 75 µg/ml collagen I-coated tissue culture plastic at confluence and allowed to attach overnight. Cells were quickly washed in phosphate-buffered saline (PBS) three times, and cells were dissociated with a non-enzymatic dissociation buffer (3 mM EDTA, 2% glycerol, 15 mM sodium citrate in PBS, pH 7.4.) and resuspended in 1% BSA in PBS. Cells were washed two times in PBS containing Ca^2+^ and Mg^2+^ (PBS-2^+^; Gibco 14040117) and treated with 5 mM NHS-PEG4-Biotin (Thermo Fisher Scientific, #A39259) for surface labeling or 5 mM BS^3^ (Thermo Fisher Scientific, #PIA39266) in PBS-2^+^ medium for 30 min at 4°C. Cell suspensions were quenched with 20 mM Tris in PBS-2^+^ for 15 min and washed with 20 mM Tris in PBS-2^+^ twice to fully quench crosslinking reactions. Cells were then washed three times in PBS-2^+^ and lysed in denaturing lysis buffer (RIPA lysis buffer plus 0.5% SDS). Biotin-labeled cells were subjected to immunoprecipitation with streptavidin beads (Sigma-Aldrich; S1638) or anti-DDR2 antibodies and were immunoblotted as described above. BS^3^ crosslinking experiments were subjected to SDS–PAGE on 6% polyacrylamide gels and immunoblotted as described above.

### Conditioned medium generation

Tumor and CAF cells were plated on collagen I-coated tissue culture plastic at 70% cell density overnight in full growth medium (described in the ‘Cell lines and lentiviral constructs’ section). Plates were washed extensively in serum-free medium (DMEM without serum and penicillin or streptomycin supplementation) to remove residual serum, and serum-free medium was added to the cells for 24 h. For some experiments, serum-free medium included DMSO (0.1%) or 10 µM CR13452. Conditioned medium was removed from cells and centrifuged at 400 ***g*** to remove cells. Conditioned medium was then concentrated through PBS-washed 10 kDa protein concentrators (Thermo Fisher Scientific) to remove peptides and any residual CR13452. Concentrated proteins were resuspended in serum-free medium and quantified, and equivalent amounts of protein were utilized to condition Matrigel and ECM collagen matrices.

### Invasion and migration assays

8 µm Transwell membranes were coated with 70 µl of 1 mg/ml GFR Matrigel, which was then allowed to solidify. For normal invasion assays, 20,000 tumor cells were added to the top of the Matrigel plug and allowed to invade for 24 h (human tumor cells) or 48 h (mouse tumor cells). For conditioned medium experiments, equal amounts (5 µg) of conditioned medium were added to the Matrigel plugs and allowed to incubate for 24 h. 20,000 tumor cells were then added to the top of the conditioned medium-treated Matrigel and allowed to invade for 24 h. Non-invaded cells were removed using a cotton swab, and then membranes with invaded cells on their underside were fixed and stained utilizing the HEMA-3 staining kit (Thermo Fisher Scientific; 122-911), following the manufacturer's protocol. 3–4 random areas were imaged by brightfield microscopy, utilizing a 10× objective, and quantified.

3D collagen migration assays were performed as previously described (Zhang et al., 2013). Briefly, 100,000 tumor cells were encapsulated in a 3D collagen I plug, transferred to a 12-well plate coated with 3D collagen, overlaid with full growth medium and allowed to migrate. Images were taken on a brightfield microscope with 4× objective every 24 h. Distance migrated was measured as the furthest distance a cell invaded away from the original boundary. For conditioned medium experiments, all 3D collagen I was formed with conditioned medium, including the 3D collagen plug containing the cells, and conditioned medium was added to the top of the gels, allowed to migrate and measured as above.

### Animal models and experiments

Balb/c and FVB/N female mice were purchased from Charles River Laboratory. The generation and characterization of *Ddr2*^−/−^ mice has been previously described (Corsa et al., 2016). The *Ddr2*^−/−^ allele has been backcrossed onto both FVB/N and Balb/c genetic backgrounds (>10 generations). MMTV-PyMT mice were purchased from Jackson Laboratories and maintained on FVB/N background. For syngeneic orthotopic transplants, 1×10^6^ 4T1 cells (Balb/c) were injected into the fourth mammary fat pad of 8-week-old Balb/c females and allowed to reach end stage (2 cm primary tumor). Primary tumors and lungs were collected at end stage and processed for immunohistochemistry as previously described (Brenot et al., 2018). Lungs were processed in three step-sections of 50 µm steps with two serial sections of each step. Metastatic tumor nodules were counted and averaged per lobe of lung (five lobes) per mouse. Syngeneic lung colonization experiments were conducted by injecting 7.5×10^5^ PyMT-derived FVB/N tumor cells with or without 1×10^5^ CAFs (from FVB/N mice) of indicated genotypes into the tail vein of 8-week-old FVB/N female mice. After 25 days the lungs were harvested and processed as above. All animal experiments were performed according to Washington University Institutional Animal Care and Use Committee under protocol #2019-1042.

### Breast tumor organoid migration in microfluidic device

Mice mammary tumor organoids were obtained as previously described (Corsa et al., 2016), mixed with 2 mg/ml collagen I solution, loaded into the middle tissue channel of a previously published microfluidic device (Hwang et al., 2019) and allowed to polymerize (37°C, 20% O_2_). After collagen polymerization, medium was delivered to the top and bottom fluidic lines and cultured in 5% O_2_ for 48 h, with medium changes every 24 h. After 48 h of culture, a gradient of conditioned medium was established by delivering conditioned medium (50 ng/μl) from the various genetically modified mouse CAFs (*Ddr2*^+/+^, *Ddr2*^−/−^, WT DDR2-rescued *Ddr2*^−/−^ and K608E-rescued *Ddr2*^−/−^). Live-cell time-lapse imaging (using a Nikon Tie2 with 63× objective and controlled temperature, humidity and 5% O_2_) was performed by taking images every 20 min for a maximum of 18 h. After imaging, analysis was performed by marking organoids and tracking cell migration using NIS-elements analysis software (Nikon). Average velocity (mm/min) and migration efficiency (directed migration) of the organoids over the entire imaging period was calculated, as described previously (Hwang et al., 2019).

### Two-photon and second harmonic generation microscopy

Collagen I solution (2 mg/ml) was loaded into the middle tissue chamber of the microfluidic device and allowed to polymerize (37°C, 20% O_2_). CAFs from various genetically modified mice (*Ddr2*^+/+^, *Ddr2*^−/−^, *Ddr2*^−/−^ rescue *Ddr2* and *Ddr2*^−/−^ rescue K608E) were mixed with 2 mg/ml collagen I solution and loaded to the chamber immediately flanking the middle chamber and allowed to polymerize. After collagen polymerization, medium was delivered to the top and bottom fluidic lines and cultured in 5% O_2_, 5% CO_2_ for 48 h with medium change every 24 h. After culture, freshly prepared paraformaldehyde was infused through the fluidic lines overnight. Second-harmonic imaging (SHG) was performed on the collagen within the CAF free chamber (middle chamber). SHG images were obtained on a Zeiss LSM 880 Airyscan with a 40× objective. After imaging, analysis was performed using CT-Fire analysis software (Bredfeldt et al., 2014). Collagen I fibers within the entire middle chamber were quantified for length (μm), width (μm), angle, and straightness. To ensure homogeneity across the entire middle chamber, it was split into four sections that were analyzed individually and cross referenced with each other; no change was observed between these sections.

### Statistical analysis

Statistical analysis was performed utilizing Prism 8.4. Figures are presented as mean±s.e.m. Invasion assays and lung metastasis assays were analyzed via one-way ANOVA with post-hoc Tukey test. Assays involving multiple comparisons were analyzed utilizing a two-way ANOVA with a mixed model comparison. *P*-values below 0.05 were considered significant. All data was generated from at least three independent experiments.

## Supplementary Material

Supplementary information

Reviewer comments
