# Supplementary material for: Tyrosine kinase-independent actions of DDR2 in tumor cells and cancer-associated fibroblasts influence tumor invasion, migration and metastasis
Source: J Cell Sci. 2021 Oct 13;134(19):jcs258431. doi: 10.1242/jcs.258431 (PMC8542384; doi:10.1242/jcs.258431)
Supplement: Supplementary information [file joces-134-258431-s1.pdf]

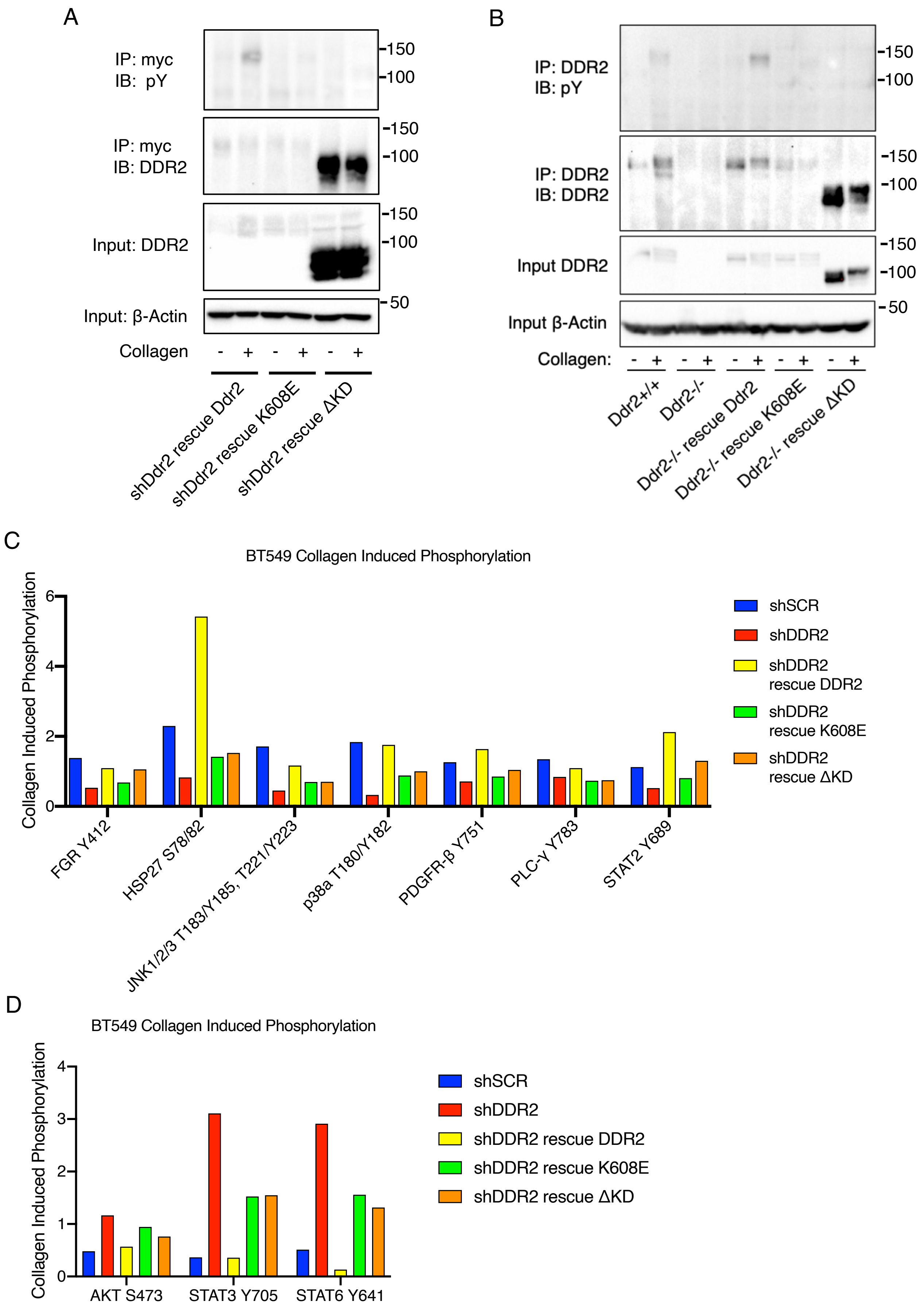

Journal of Cell Science • Supplementary information

Fig. S1. Supplemental data to Figure 1. Collagen-induced phosphorylation of cellular signaling mediators. **(A and B)** Tyrosine phosphorylation of DDR2 rescue constructs. shDdr2 knockdown-rescue 4T1 cells **(A)** or mouse CAFs **(B)** were plated +/- collagen-I coated plates for 6 h. DDR2 was immunoprecipitated with anti-myc (4T1 cell lines) or anti-DDR2 (mouse CAF) antibodies and bound products Western blotted with anti-P-Tyr antibody. Input controls are 10% of total lysate used for IP. **(C and D)** BT549 cells of the indicated DDR2 genotype were cultured on 2 mg/ml collagen-I gels for 6h, lysed, and processed utilizing the manufacturer protocol for human phospho-kinase array (R&D Systems ARY003C). **(C)** DDR2-dependent increase in phosphorylation. **(D)** DDR2-dependent decrease in phosphorylation.

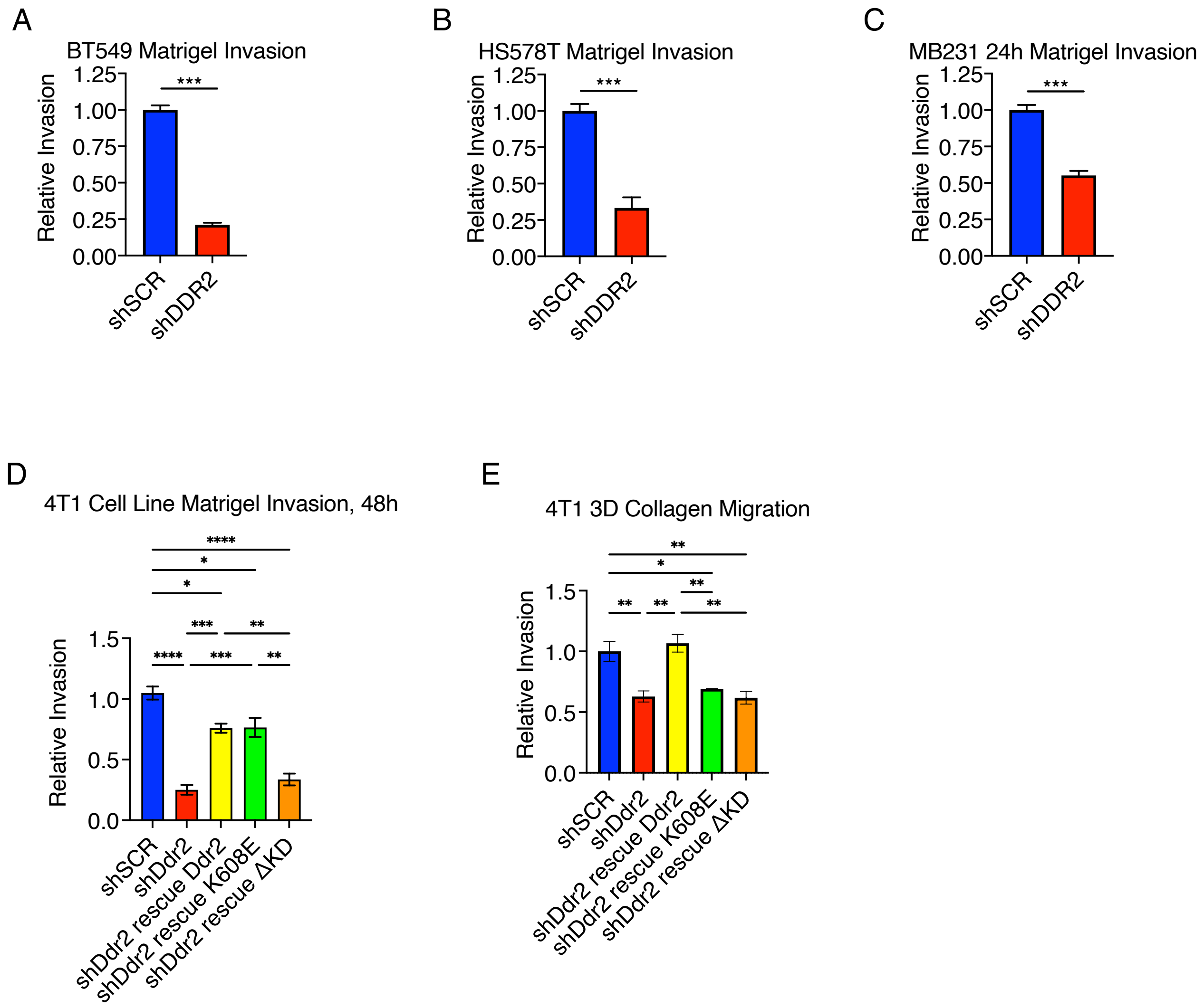

Fig. S2. Supplemental data to Figure 2. Tumor cell invasion is impeded by loss of DDR2. **(A)** BT549 cells of indicated genotype plated on Matrigel and allowed to invade for 24h, relative comparison to shSCR. Statistics: ***p<0.001, t-test. **(B)** HS578T cells plated as in A, relative comparison to shSCR. Statistics: ***p<0.001, t-test. **(C)** MDA-MB-231 cells plated as in A, relative comparison to shSCR. Statistics: ***p<0.001, t-test. **(D)** 4T1 cells plated as in A allowed to invade 48h, relative comparison to shScr. Statistics: *p<0.05, ***p<0.001, one-way ANOVA. **(E)** 4T1 cells plated in 3D collagen matrices and allowed to invade for 72h. Relative invasion to shSCR cells shown. Statistics: *p<0.05, **p<0.01, one-way ANOVA

Journal of Cell Science • Supplementary information

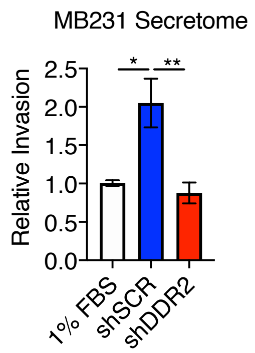

Fig. S3. Supplemental data to Figure 3. Human tumor cell conditioned media invasion through Matrigel. Matrigel plugs were treated with conditioned media from MDA-MB- 231 cell of the indicated genotype for 24h, MDA-MB-231 cells were added and allowed to invade for 24h, relative comparison to unconditioned Matrigel. Statistics: *$p<0.05$, **$p<0.01$, one-way ANOVA.

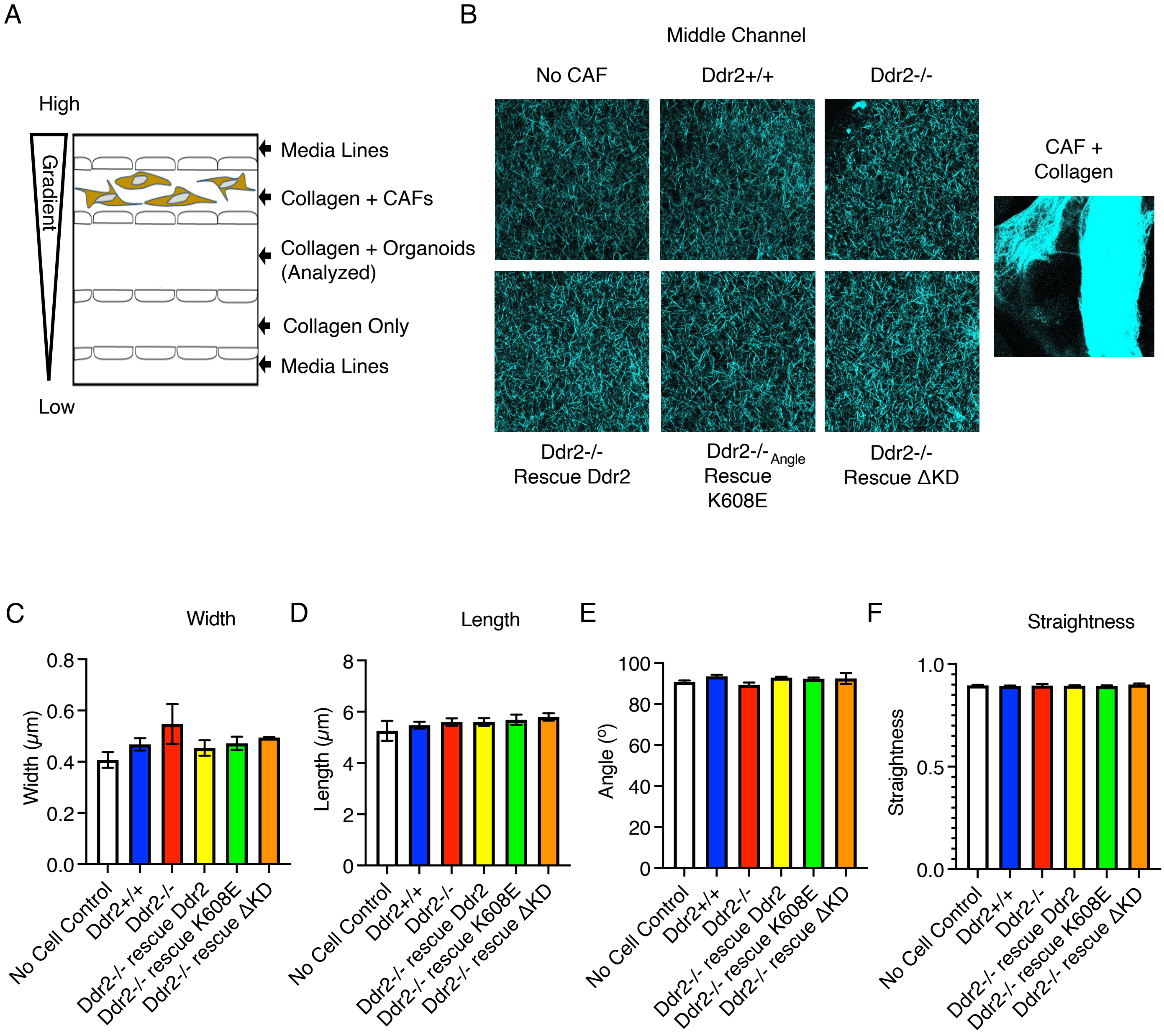

Fig. S4. CAFs generating conditioned media do not alter collagen fiber characteristics in the microfluidic channel where primary tumor organoids migrate. (A) Microfluidic device setup and analysis key. Only devices in which CAFs did not enter the center channel were analyzed. **(B)** Representative images of collagen in the central analysis channel (left) or CAF+collagen channel (right). **(C-F)** Width **(C)**, Length **(D)**, Angle **(E)**, and straightness **(F)** of collagen fibers imaged via second harmonic generation microscopy and analyzed via ct-FIRE/Curvealign software. No statistical difference is noted for any collagen fiber characteristic, one-way ANOVA.
