# Supplementary material for: Tyrosine kinase-independent actions of DDR2 in tumor cells and cancer-associated fibroblasts influence tumor invasion, migration and metastasis
Source: J Cell Sci. 2021 Oct 13;134(19):jcs258431. doi: 10.1242/jcs.258431 (PMC8542384; doi:10.1242/jcs.258431)
Supplement: Reviewer comments [file joces-134-258431_review_history.pdf]

Journal of Cell Science

Tyrosine kinase-independent actions of DDR2 in tumor cells and cancer-associated fibroblasts influence tumor invasion, migration and metastasis

Craig E. Barcus, Priscilla Y. Hwang, Vasilios Morikis, Audrey Brenot, Patrick Pence, Maria Clarke and Gregory D. Longmore
DOI: 10.1242/jcs.258431

Editor: Kathleen Green
Review timeline

Original submission:	19 January 2021
Editorial decision:	25 February 2021
First revision received:	20 July 2021
Editorial decision:	11 August 2021
Second revision received:	20 August 2021
Accepted:	23 August 2021

Original submission

First decision letter

MS ID#: JOCES/2021/258431

MS TITLE: Tyrosine kinase independent actions of DDR2 in breast tumor cells and breast tumor stromal CAFs influence invasion and metastasis

AUTHORS: Craig E Barcus, Priscilla Y Hwang, Audrey Brenot, Patrick Pence, and Gregory D. Longmore
ARTICLE TYPE: Research Article

We have now reached a decision on the above manuscript.

To see the reviewers' reports and a copy of this decision letter, please go to: https://submit-jcs.biologists.org and click on the 'Manuscripts with Decisions' queue in the Author Area. (Corresponding author only has access to reviews.)

As you will see, the reviewers raise a number of substantial criticisms that prevent me from accepting the paper at this stage. They suggest, however, that a revised version might prove acceptable, if you can address their concerns. In addition to the reviews, we also received some feedback after making the reviews accessible for discussion among the referees. Things identified as part of this process, and that required additional experimentation are:

Western blots validating DDR2 knockdown and rescue efficiency for the additional cell lines (Hs578T, MDA-MB-231, 4T1). At a minimum there should be a Western blot of whole cell lysates and phospho-tyrosine IB:IP as in Fig. 1C to ensure that KD is substantial and kinase activity is gone in mutant cell lines. This is particularly important for 4T1 cells which are used in in vivo studies.

A number of conclusions differentiating kinase activity from the presence of the kinase domain in Figs. 2-4 rest on data provided from a single cell line as described in the reviews. This needs to be addressed with experiments in additional cell lines if the data is not already available.

© 2021. Published by The Company of Biologists under the terms of the Creative Commons Attribution License (https://creativecommons.org/licenses/by/4.0/).

One point raised by reviewer #2, which was not noted by the other reviewers is eliminating the possibility of a direct effect of CR 13452 on cell invasion vs an indirect effect on CAF CM. You could address this by deleting that experiment entirely. Alternatively, additional experiments would be necessary to address this as described.

Finally, comments from referee #2 about Fig. 7 might be addressable through citing additional literature. You could comment in the discussion about some of the uncertainties about where and how CAFs might be supporting enhanced metastatic colonization in this model and not address these in vivo studies with additional experiments.

If you think that you can deal satisfactorily with the criticisms on revision, I would be pleased to see a revised manuscript. We would then return it to the reviewers.

We are aware that you may be experiencing disruption to the normal running of your lab that makes experimental revisions challenging. If it would be helpful, we encourage you to contact us to discuss your revision in greater detail. Please send us a point-by-point response indicating where you are able to address concerns raised (either experimentally or by changes to the text) and where you will not be able to do so within the normal timeframe of a revision. We will then provide further guidance. Please also note that we are happy to extend revision timeframes as necessary.

Please ensure that you clearly highlight all changes made in the revised manuscript. Please avoid using 'Tracked changes' in Word files as these are lost in PDF conversion.

I should be grateful if you would also provide a point-by-point response detailing how you have dealt with the points raised by the reviewers in the 'Response to Reviewers' box. Please attend to all of the reviewers' comments. If you do not agree with any of their criticisms or suggestions please explain clearly why this is so.

Reviewer 1

Advance summary and potential significance to field

In this work, Barcus et al describe the role of DDR2 in breast cancer invasion and metastasis, focusing on the kinase independent effects of this receptor.
Using different in vitro and in vivo approaches, they demonstrate that, apart from the specific kinase activity of DDR, the kinase domain itself can regulate cell migration and invasion. They show that DDR2 expression, and its kinase domain, affect invasion through paracrine signalling, not only in cancer cell cultures, but also when cancer cells are cocultured with CAFs lacking or not kinase activity/kinase domain in DDR2. The work is of potential interest in the field of cancer therapeutics as it opens the door to target the tyrosin kinase independent activity of DDR2.

Comments for the author

Even if the methodological approaches are nicely designed, this reviewer feels that in general the claims that the authors make through the manuscript are not entirely supported by the data. In some experiments the authors find that mutating the kinase domain can still somehow promote invasion compared with ΔKD condition, but the effects of the K608E mutant compared with control are rather limited. DDR2 K608E mutant cells still have slight levels of pTyr compared to ΔKD, which might explain those differences. So, in summary, this reviewer is not entirely convinced that there's no kinase activity in the K698E mutants, and thus the authors should turn down their claims throughout the manuscript.
Apart from this, I have some major questions regarding the experiments:
-1. The main claim of the paper (and the most interesting from my point of view) is that DDR2 control invasion from a paracrine mode, both from cancer cells and from CAFs. However, even if understanding which secreted molecule(s) could be driving invasion depending on the status of DDR2 is probably the most outstanding question here, this has not been addressed by the authors. It would be very interesting to perform, for example, mass spectrometry-based secretome analysis for this. However, I am aware of the workload of this type experiments.

© 2021. Published by The Company of Biologists under the terms of the Creative Commons Attribution License (https://creativecommons.org/licenses/by/4.0/).

-2. Figure 4: It would be interesting to see the ability of metastasis formation of ΔKD cells, to be able to compare this to K608E.
-3. Figure 5. The authors use a nice microfluidic device to check chemotaxis and directional migration. However, I have several important concerns with this figure:
- Even if authors say that in experiments using this device they only take into account those where CAFs do not reach cancer cells to avoid physical contact effects, CAFs might be pulling collagen fibers from the lateral chamber generating directional cues for the cancer cells to migrate. Taking into account that the authors previously reported that DDR2 affects force generation, I think that this is indeed a very likely possibility. To discard this possibility the authors should analyze collagen alignment in the different conditions (software like CurveAlign can be useful for this). Also, it would be important to analyze single CAF traction forces for each condition, and correlate this with collagen alignment and cancer cells invasion.
- The authors claim that cancer cells invade through collagen collectively. To show this the authors need to perform stainings of migrating cells for cell-cell junction markers, for example.

Minor points
- It would be interesting to know the cellular localization of SNAIL1 in the different experimental conditions.
- I would suggest generating polar histograms for representation of the directional migration.
- I suggest that the authors unify some plots to make it easier for the readers. For example, why not putting figure 2A and B together? There are many other examples like this throughout the figures.
- It would help the readers adding a schematic model of DDR2 actions in CAFs/Cancer cells.

Reviewer 2

Advance summary and potential significance to field

Demonstration that kinase independent functions of DDR2 support breast cancer metastasis.

Comments for the author

In JOCES:2021:258431 Barcus et al. provide evidence that DDR2 supports metastatic phenotypes both directly and through paracrine effects mediated by CAFs in a manner that is not entirely dependent on the tyrosine kinase activity of DDR2. In principle this work provides an important new direction to role of DDR2 in breast cancer metastasis from the Longmore lab and others and would be of interest to the field as it would support efforts to understand kinase-independent functions of DDR2. How a kinase independent function of DDR2 affects breast cancer cells directly or indirectly influences the secretome of CAFs is not made clear here, but this is not considered a necessary element for publication. Generally, the study employs a rigorous experimental approach involving shRNA-mediated DDR2 knockdown complemented with WT, kinase inactive and kinase domain deleted mutants and attemtes to use multiple mouse and human breast cancer cell lines to test the role of the kinase activity and kinase domain of DDR2. However, there are a number of instances in which the rigor of this overall approach was not uniformly applied resulting in inadequate support for key findings. If these are addressed it would make this work suitable for publication in the Journal of Cell Science.

Major issues:
Western blots validating DDR2 knockdown and rescue efficiency for the additional cell lines (Hs578T, MDA-MB-231, 4T1) employed beyond BT549 shown in Fig.1 should be shown in a supplemental figure as these are employed elsewhere in the paper. 4T1 is shown in Fig. 4A but this cell line and its derivatives are used in prior figures so a Supplemental Figure showing all of the additional engineered cell lines would seem the most economical way to present the data. It is not necessary to perform all of the assays in Fig. 1 for all of the cell lines, however, at a minimum there should be a Wesetrn blot of whole cell lysates and phospho-tyrosine IB:IP as in Fig. 1C to ensure that KD is substantial and kinase activity is gone in mutant cell lines. This is particularly important for 4T1 cells wich are used in in vivo studies.
This sentence (lines 151-53) is not supported by the data: “In both human (Fig. 2A-C) and mouse breast tumor cells (Suppl. Fig. S2D), the cytoplasmic tyrosine kinase domain, but not tyrosine

© 2021. Published by The Company of Biologists under the terms of the Creative Commons Attribution License (https://creativecommons.org/licenses/by/4.0/).

kinase activity, was required for Matrigel invasion." Fig. S2D does not show data for the kinase domain deleted mutant. Thus, this important conclusion apparently rests on only one cell line. The data for other cell lines should be included.
Moreover, the collagen I invasion migration assay (Fig. 2D-F) is shown for a single cell line, limiting the conclusion that can be drawn here. In fact it looks like the kinase domain deleted mutant has less invasion than the knockdown alone (Fig. 2E) but this is not commented on.
Again, the kinase domain deleted mutant is only tested in BT549 cells in Fig. 3.
This sentence (lines 170-71) is not clearly supported by the data: "The increase in Matrigel invasion induced by conditioned media also required the presence of DDR2 in tumor cells." shSCR control cells should be tested in the same experiment alongside shDDR2 cells to support this claim.
Why not the kinase deletion mutant in Fig. 4?
For the experiment in Fig. 5E,F, the authors have not excluded the possibility that the small molecule DDR2 inhibitor is present in the CM of treated samples and thereby directly inhibiting invasion, a trivial explanation rather than indirectly via its effects on CM derived from CAFs as proposed. The direct effect of CR13452 on CM stimulated migration BT549 cells can be measured by including it in the invasion assay. If there is a significant direct effect of CR13452, can the authors dialyse the CM through a low MW cutoff and/or measure the levels of CR13452 in the CM via mass spec? Otherwise this experiment could be omitted without jeopardizing the overall MS.
For Fig. 7, is there any precedent for this experiment, e.g. have CAFs been co-injected with tumor cells in a tail vein injection metastatic colonization model? If so, this work should be cited. If not, this experiment raises some interesting questions that should be explored. Are the CAFs present in metastatic colonies? If not, where are they and how plausible is it that a relatively small number of CAFs (10e5) could exert paracrine effects systemically? Must the CAFs be co-injected into the tail vein or could they be implanted subcutaneously and exert the same effect on lung colonization of PyMT tumor cells?

Minor Issues:
MW markers on Fig. 1B. How were Western blot signals quantified?
Address discrepancy in differences in levels of DDR2 detected in Fig. 1B and Fig. 1E between shSCR and shDDR2 + rescue constructs. Fig.1E looks much more than the 3-4 fold difference claimed for Fig. 1B. Does this suggest that overexpressed mutant DDR2 rescue constructs are trafficking to the surface much more efficiently than endogenous DDR2? What about WT DDR2 rescue in this assay?
This sentence (lines 141-42) is puzzling in light of the data presented in Fig. S1: "These analyses suggested that in all cell lines there was a quantitative difference in signaling, but no unique or qualitative signaling differences." The authors should clarify their interpretation of this data. Given the limits of this experiment and that none of the observations from the array were validated further, this data might be removed as it does not advance the story.
For Fig. 6, the authors should explain in the methods how collective migration of tumor organoids is quantitated (perhaps seperately from other modes of migration?). This is not made apparent in the cited reference #3.
For the discussion a couple of additional points could be considered: 1) Two alternatively spliced isoforms of DDR1 have been described (DDR1d and DDR1e) that are truncated or lack kinase activity. Has there been any effort to identify similar alternative spliceforms for DDR2? Does this also support kinase independent functions in the DDR family? 2) Any speculation on how DDR2 might be programming the secretome of CAFs?

Reviewer 3

Advance summary and potential significance to field

The study of Barcus et al focus on the collagen kinase receptor, DDR2 (Discoidin Domain Receptor 2) in breast cancer, a key receptor in mediating tumor cell-collagen interactions. The authors demonstrate that both tumor- and stroma-associated DDR2 can elicit pro-invasive effects on breast cancer cells independent of its kinase activity. These important findings help to define the action of this receptor in breast cancer malignancy and the strategies to target its effects in cancer patients. Overall, this is a very well-conducted rigorous and novel study with solid data that supports the hypothesis and brings new and important information to the field.

© 2021. Published by The Company of Biologists under the terms of the Creative Commons Attribution License (https://creativecommons.org/licenses/by/4.0/).

Comments for the author

This reviewer does not request additional experimentation, just addressing the comments and some issues with data interpretation as well as expanding the discussion, if possible.

Specific points
1. Please can the authors add molecular weight markers to all the blot panels?
2. Fig. 1A. Please indicate what are the different shapes intend to represent in the cartoon of DDR2 domains.
Please also provide the last amino acid of DDR2 tagged with Myc. Please explain why was important to delete the entire KD also to address kinase function when compared to the point mutant kinase inactive.
1B. Why is ERK1/2 shown, as a loading control? Seems odd considering could be regulated by collagen signaling, even total ERK. Please indicate in the legend that is a loading control. The legend states: “probed with the indicated antibodies” but none are indicated in 1B. Indicate the mass and what are the different species detected in that blot.

1D. Please label the DDR2 forms detected in the blot. Is unclear in this Figure whether collagen promotes oligomerization. Does it? If available, the authors could also show a blot of the parental cells with endogenous DDR2 under the same conditions, to demonstrate that their recombinant system reliably reflect the behavior of the natural endogenous receptor. Also, is unclear why such a long time (overnight) culturing on collagen was chosen as a method to stimulate receptor activation. Could this long time decrease total levels of receptors due to turnover/endocytosis? In other experiments a 6 h time of incubation was used.
1E. Lacks the delta KD construct.
The authors mentioned several breast cancer cell lines, but the data on expression and signaling focuses mostly on BT549. Is it the same in the other cell lines, even signaling? If no data are mentioned for the others (even as not shown), then please remove from the manuscript or precisely describe what cell lines were used in each experiment. If available, do the authors have information on DDR1 expression (or its potential induction by the culture on collagen, matrigel or expression of DDR2 constructs, or the conditioned media) in any of the cell lines used, including 4T1?
3. Supp Fig 1. The phosphorylation array uses a different collagen concentration and status (gel) than the experiments used to establish the expression and activation of the constructs, can you provide an explanation why? Is it expected the system/constructs to behave in a similar fashion (extent of activation, surface amounts, multimerization, turnover) under 2D conditions? These data also lack no collagen, so could results represent collagen effects independent of DDR2? How significant are the differences observed? The delta KD and the K608E mutant appear to behave the same (see point 2).
The conclusion of this study states that “these analyses suggested that in all cell lines there was a quantitative difference in signaling, but no unique or qualitative signaling differences”. Regardless, this conclusion/data interpretation is unclear or written in a convoluted manner. Were there or were there no differences? Please be more specific/clear. But what does it mean?
4. Suppl. Fig. 2 and Fig 2A/B. Matrigel, which contains collagen IV, is used to assess invasion. Yet, DDR2 is a fibrillar collagen receptor and DDR2 supports invasion through this matrix. This is a very interesting part of the study because it may imply two different modes of action of DDR2 mediating invasion of basement membrane matrices and interstitial collagen matrices. Based on the data shown, the kinase mutant rescues invasion of Matrigel in the DDR2-silenced cells (2A) but not the truncated KD (2B). These data suggest that kinase activity is required for the pro-invasive action of DDR2 on Matrigel but a drastic truncation of the KD disrupts the kinase activity independent effect of the receptor on this matrix. While in the Discussion (lines 346-352) the authors discuss invasion of 3D collagen I, the Matrigel data are not discussed. Yet, it is nicely shown that conditioned medium from cells with reduced DDR2 inhibit invasion, suggesting that in this medium there is an inhibitor or lacks a stimulator of Matrigel invasion. Moreover, media from cells expressing the kinase defective DDR2 but not the wild type receptor recovers some of the invasive activity. However, the data interpretation of these interesting experiments in the Discussion is not well developed and hardly discusses how DDR2 can promote invasiveness of Matrigel in a kinase independent manner. Any idea/speculation what these media may contain based on the array data (Supp Fig 1)? Did the author test whether a DDR2 kinase inhibitor or neutralizing antibodies (if available) have an impact in these assays?

© 2021. Published by The Company of Biologists under the terms of the Creative Commons Attribution License (https://creativecommons.org/licenses/by/4.0/).

Why the conditioned media were obtained from cells cultured on collagen (line 449) and not on Matrigel too?
The concentration of collagen used is not indicated also.

Can the authors speculate how kinase inactive DDR2 supports breast cancer invasion of a basement membrane-like matrix? Any ideas? Is it known whether breast cancer cells within the confines of a basement membrane (in situ carcinoma) express DDR2 in human tissues?
Could the findings in Fig 1 be different in terms of DDR2 regulation when cells are seed on Matrigel? Do the authors know how the DDR2 constructs behave (surface expression, oligomerization) when the same cells are seed on Matrigel-coated dishes, in similar experiments to those described in Fig. 1 (and Methods section line 435).
Are cells expressing the delta KD attaching more rapidly/strongly to collagen?
5. Fig. 4C can be interpreted that kinase activity contributes to the metastatic ability of the 4T1 cells because the KD mutant fails to restore full metastatic ability when compared to the shSCR or wild type DDR2 and there is no indication of the levels of the various DDR2 forms in the tumor cell in vivo. However, in spit that this is a great experiment, data interpretation appears a bit bias and could be improved by more precise language.
6. The authors argue their assay in Fig. 6 discriminate between single and collective migration. However, this is not explained in the results or method section. This should be described for the general reader.
7. Again, conditioned media seems to be a critical mediator of DDR2 action also in DDR2-manipulated CAFs.
Can the authors discuss/speculate what could be the mechanism/factor?

Minor points:
Line 109: “as determined by DDR2 immunoprecipitation total phosphotyrosine immunoblot” should read as determined by total DDR2 immunoprecipitation followed by immunoblot analyses for phosphotyrosine residues.
Line 348-9. The sentence: “Possibly cells expressing DDR2.DKD interact more avidly with collagen in part because they cannot be endocytosed” should replace by: because these truncated receptors cannot….
Otherwise, they refer to the cells.
Line 352: the dominant inhibitory manner the authors mention is unclear. What could the target be of the dominant effect? Apparently, there is no wild type DDR2. Perhaps this sentence (lines 351-352) should be followed by the reasoning described on line 353, without starting a new paragraph.
Fig 2C and 2F could be supplemental.
Line 144. The authors use migration/invasion as co-dependent mechanisms. While invasion may require cell migration, highly migratory cells may not necessarily be invasive. For instance, lack of a productive proteolytic system may compromise matrix penetration. Therefore, because the authors have not determined effects on migratory activity and their assay supposedly reflects invasion, I suggest removing the
migration/invasion term.

First revision

Author response to reviewers' comments

We thank the referees for their thoughtful critiques, and the opportunity to respond and submit a revised manuscript. In doing so we have done a number of new experiments, both in cell based assays with another cell line and in vivo. No doubt the suggested revisions and added experiments have strengthened the quality and hopefully suitability of our revised manuscript. Our point-by-point responses (black) to all of the reviewer’s concerns (blue) are below. New data and textual revisions are highlighted.

Editor General Discussion Points:
Western blots validating DDR2 knockdown and rescue efficiency for the additional cell lines

© 2021. Published by The Company of Biologists under the terms of the Creative Commons Attribution License (https://creativecommons.org/licenses/by/4.0/).

(Hs578T, MDA-MB-231, 4T1). At a minimum there should be a Western blot of whole cell lysates and phospho-tyrosine IB:IP as in Fig. 1C to ensure that KD is substantial and kinase activity is gone in mutant cell lines. This is particularly important for 4T1 cells which are used in in vivo studies.

This has now been done for human BT549 and mouse 4T1 breast tumor cell lines as well as with mouse breast CAFs. Moreover most Matrigel invasion and collagen migration assays have now been done with both BT549 and 4T1 cells. In addition, secretome assays have been done with BT549, 4T1, mouse CAFs, and human CAFs. Throughout results are similar in all cell lines.

A number of conclusions differentiating kinase activity from the presence of the kinase domain in Figs. 2-4 rest on data provided from a single cell line as described in the reviews. This needs to be addressed with experiments in additional cell lines if the data is not already available.

This has now been done throughout the manuscript (figures) with the mouse breast tumor cell line 4T1. In addition, secretome analyses were also performed in CAFs expressing the same panel of DDR2 mutants.

One point raised by reviewer #2, which was not noted by the other reviewers is eliminating the possibility of a direct effect of CR 13452 on cell invasion vs an indirect effect on CAF CM. You could address this by deleting that experiment entirely. Alternatively, additional experiments would be necessary to address this as described.

We chose to keep this data, as CR13452 is a unique DDR2 inhibitor. We have performed new control experiments to address reviewer 2 concern. These new control experiments are presented in Supplemental Fig. S4. Moreover, we have highlighted in methods and results that all culture supernatants analyzed were filtered through a 10 kDa cut-off to remove peptides and any residual CR13452 (see new Fig. 5G).

Finally, comments from referee #2 about Fig. 7 might be addressable through citing additional literature. You could comment in the discussion about some of the uncertainties about where and how CAFs might be supporting enhanced metastatic colonization in this model and not address these in vivo studies with additional experiments.

We chose to address this experimentally. We labelled CAFs with RFP and repeated the experiment. At 2 days after IV injection, injected RFP+ CAFs are present in the lungs, however, by day 7 there were few present. Tumors were scored on day 7. This result suggests that at least early on CAFs could be contributing to the observed outcome.

Reviewer 1:

General Comment:

Even if the methodological approaches are nicely designed, this reviewer feels that in general the claims that the authors make through the manuscript are not entirely supported by the data. In some experiments the authors find that mutating the kinase domain can still somehow promote invasion compared with ΔKD condition, but the effects of the K608E mutant compared with control are rather limited. DDR2 K608E mutant cells still have slight levels of pTyr compared to ΔKD, which might explain those differences. So, in summary, this reviewer is not entirely convinced that there's no kinase activity in the K698E mutants, and thus the authors should turn down their claims throughout the manuscript

We assessed tyrosine autophosphorylation of the cytoplasmic domain of DDR2 by its own cytoplasmic tyrosine kinase domain in now 3 cell lines: human BT549 (Fig. 1), mouse 4T1 (Suppl. Fig. S1A), and mouse breast CAFs (Suppl. Fig. S1B). This was done in WT, K608E, and ΔKD DDR2 expressing lines. While there is the possibility of a small amount of residual WT DDR2 in BT549 and 4T1 cells as these were generated in shRNA knockdown cells (never 100% depletion), in mouse CAFs these are genetically DDR2 null cells. In all instances we do not observe any detectable tyrosine phosphorylation of DDR2 in K608E and ΔKD expressing cells. Strictly speaking there could still be other "kinase" activities associated with these mutants in cells. But our intent was to determine if inhibiting the tyrosine kinase activity of DDR2 clinically with TKIs, such as dasatinib (in use clinically), actually inhibit DDR2 actions in cells. We believe we have shown both in cell culture

© 2021. Published by The Company of Biologists under the terms of the Creative Commons Attribution License (https://creativecommons.org/licenses/by/4.0/).

cancer assays (invasion and migration) and in vivo (lung metastases) that inhibition of the tyrosine kinase activity of DDR2 alone is not sufficient. In other words there is residual tyrosine kinase-independent activity for DDR2. What that activity is remains to be determined. In contrast, removal of the tyrosine kinase domain appears to completely inhibit all actions of DDR2 in cells and in vivo. Of note, the CR13452 inhibitor we used herein is NOT a TKI. It is an allosteric regulator of DDR2 that acts via the extracellular domain (Grither and Longmore, PNAS 2018). It appears to be more potent than either the K608E mutant or TKI inhibitors – equivalent to the genetic null cells.

The cytoplasmic tail truncation removes the entire tyrosine kinase domain, while retaining the intracellular juxta-membrane domain critical for receptor dimerization and collagen binding. There are a couple of tyrosine residues in the juxta membrane region. The ΔKD mutant was chosen as it will also be tyrosine kinase inactive but allows us to assess whether cytoplasmic signaling adapters recruited to this domain (in the absence of tyrosine phosphorylation) are important if tyrosine kinase independent actions of DDR2 are apparent (which we do observe).

Despite all these arguments we have tried to tone down conclusions throughout, as well as point out the possibility of undetectable tyrosine kinase activity in the BT549 and 4T1 cells (shRNA generated) but not likely in the CAFs as they are genetic nulls.

Major Concerns:

1. The main claim of the paper (and the most interesting from my point of view) is that DDR2 control invasion from a paracrine mode, both from cancer cells and from CAFs. However, even if understanding which secreted molecule(s) could be driving invasion depending on the status of DDR2 is probably the most outstanding question here, this has not been addressed by the authors. It would be very interesting to perform, for example, mass spectrometry-based secretome analysis for this. However, I am aware of the workload of this type experiments.

We have performed multiple proteomic analyses of the secretome from CAFs (our focus going forward). We have identified a set of DDR2-regulated collagen modifying proteins as well as various cytokines. This forms the basis for subsequent efforts (ongoing), which will require substantial more work to validate and explore functional relevance in vitro and in vivo. In this manuscript we have tried to present the basis for exploring the DDR2-regulated CAF and tumor cell secretome and more importantly that tyrosine kinase inactive DDR2 appears to have preserved activity in cell based culture assays and in vivo. There is already a tremendous amount of data included in the present manuscript and now using multiple cell lines – both tumor cells and CAFs. We respectfully argue that the addition of more data, while interesting and being pursued, is beyond the scope of the present manuscript.

2. Figure 4: It would be interesting to see the ability of metastasis formation of ΔKD cells, to be able to compare this to K608E.

This has now been done in new in vivo experiments (see revised Figure 4).

3. Figure 5. The authors use a nice microfluidic device to check chemotaxis and directional migration. However, I have several important concerns with this figure:

- Even if authors say that in experiments using this device they only take into account those where CAFs do not reach cancer cells to avoid physical contact effects, CAFs might be pulling collagen fibers from the lateral chamber, generating directional cues for the cancer cells to migrate. Taking into account that the authors previously reported that DDR2 affects force generation, I think that this is indeed a very likely possibility. To discard this possibility the authors should analyze collagen alignment in the different conditions (software like CurveAlign can be useful for this). Also, it would be important to analyze single CAF traction forces for each condition, and correlate this with collagen alignment and cancer cells invasion.

-The authors claim that cancer cells invade through collagen collectively. To show this the authors need to perform stainings of migrating cells for cell-cell junction markers, for example.

We have now analyzed collagen fiber remodeling in our microfluidic devices containing the various CAF cell lines (new Supplemental Figure 4). While WT CAFs indeed remodel collagen fibers in the upper channel where they were placed, we do not detect any significant remodeling of

© 2021. Published by The Company of Biologists under the terms of the Creative Commons Attribution License (https://creativecommons.org/licenses/by/4.0/).

collagen fibers in the center of the middle channel where tumor organoids are present. So if mechanical forces are contributing, they are likely small in this experimental setup.

As to collective invasion by tumor organoids in our microfluidic device this was well-described in our Cancer Research publication from 2019. Regardless, we have now included an image of organoid migration in experiments done herein (revised Figure 6, new panel E).

In addition, we have included for your review an image of a tumor organoid migrating in our device in hypoxia and in response to an SDF1 chemical gradient. It is stained with Keratin 14 (Green) to mark the leader cells and Cdh 1 (E-Cadherin) (Red) to mark cell-cell junctions of the follower cells. Leader cells express little E-Cadherin.

NOTE: We have removed unpublished data that had been provided for the referees in confidence.

Minor Concerns:

-It would be interesting to know the cellular localization of SNAIL1 in the different experimental conditions.

We have shown in multiple other publication that collagen-DDR2 mediated stabilization of Snail1 is related to its nuclear accumulation, and thus, avoidance of cytoplasmic ubiquitination and proteosomal degradation (Nature Cell Biology 2012; J Cell Sciences 2016). We have observed this in tumor cells as well as CAFs. That said, we have not, strictly speaking, determined whether the small amount of Snail1 stabilized in K608E cells is in the nucleus.

-I would suggest generating polar histograms for representation of the directional migration.

This is a part of our analysis of leader cell polarization and directed collective migration (see Cancer Research paper 2019). Due the large number of panels in the new revised Figure 6 we have not included it, but it is referenced in the methods section.

-I suggest that the authors unify some plots to make it easier for the readers. For example, why not putting figure 2A and B together? There are many other examples like this throughout the figures.

Throughout the revised manuscript we have tried to tighten up data presentation for unity. As to the specific concern of old Figures 2A and 2B. These are different experiments, done on different days, as new cell lines were generated. As a result, we have chosen not to combine different experiments into one master figure. Sorry if this has led to some confusion or redundancy, but it seems to be the appropriate way to present our data.

-It would help the readers adding a schematic model of DDR2 actions in CAFs/Cancer cells.

Reviewer 2:

Major Concerns:

1. Western blots validating DDR2 knockdown and rescue efficiency for the additional cell lines (Hs578T, MDA-MB-231, 4T1) employed beyond BT549 shown in Fig.1 should be shown in a supplemental figure as these are employed elsewhere in the paper. 4T1 is shown in Fig. 4A but this cell line and its derivatives are used in prior figures so a Supplemental Figure showing all of the additional engineered cell lines would seem the most economical way to present the data. It is not necessary to perform all of the assays in Fig. 1 for all of the cell lines, however, at a minimum there should be a Western blot of whole cell lysates and phospho-tyrosine IB:IP as in Fig. 1C to ensure that KD is substantial and kinase activity is gone in mutant cell lines. This is particularly important for 4T1 cells wich are used in in vivo studies.

This has now been done. All constructs have been expressed in human BT549 cells (Figure 1), mouse 4T1 cells (Suppl. Fig. 1A), and mouse CAFs (Suppl. Fig. S1B). Expression level and tyrosine kinase activity has been assessed for all. Moreover, both BT549 and 4T1 cell lines have now been used for all cell based invasion and migration assays throughout the manuscript.
Importantly, results from both are equivalent. We have only analyzed CAFs secretome activity for

© 2021. Published by The Company of Biologists under the terms of the Creative Commons Attribution License (https://creativecommons.org/licenses/by/4.0/).

all mutants in human and mouse CAFs.

2. This sentence (lines 151-53) is not supported by the data: “In both human (Fig. 2A-C) and mouse breast tumor cells (Suppl. Fig. S2D), the cytoplasmic tyrosine kinase domain, but not tyrosine kinase activity, was required for Matrigel invasion.” Fig. S2D does not show data for the kinase domain deleted mutant. Thus, this important conclusion apparently rests on only one cell line. The data for other cell lines should be included.

In new experiments these experiments have been done in both human BT549 (revised Fig. 2B) and 4T1 cells (Suppl. Fig. S2D). Results in both cell lines were equivalent.

3. Moreover, the collagen I invasion migration assay (Fig. 2D-F) is shown for a single cell line, limiting the conclusion that can be drawn here. In fact it looks like the kinase domain deleted mutant has less invasion than the knockdown alone (Fig. 2E), but this is not commented on.

This has now been done in 2 cell lines: human BT549 (Fig. 2D-F) and mouse 4T1 (new Suppl. Fig S2E) tumor cell lines with similar results. ΔKD rescued BT549 cells have less activity than BT549 Ddr2-depleted, while there were no differences in 4T1 cells. This could reflect dominant inhibitory activity of ΔKD as we are using shRNA knockdown cells and depletion of WT Ddr2 is not 100% (formation of heterotypic wt DDR2 and ΔKD dimers which would be predicted to not signal). Indeed overexpression of ΔKD in parental cells (expressing DDR2) are also inhibited – which would be consistent with dominant inhibitory activity of ΔKD vis a vis wt DDR2. Importantly, however, is the fact that we do not observe any dominant inhibitory action of DKD when expressed in genetic Ddr2-/- CAFs. We have expanded this issue of the revised discussion.

4. This sentence (lines 170-71) is not clearly supported by the data: “The increase in Matrigel invasion induced by conditioned media also required the presence of DDR2 in tumor cells.” shSCR control cells should be tested in the same experiment alongside shDDR2 cells to support this claim.

This experiment, result, and sentence have been removed as it is moot. If cells lack DDR2 they do not invade, regardless of the experimental conditions.

5. Why not the kinase deletion mutant in Fig. 4?

This has now been done. See revised Figure 4

6. For the experiment in Fig. 5E,F, the authors have not excluded the possibility that the small molecule DDR2 inhibitor is present in the CM of treated samples and thereby directly inhibiting invasion, a trivial explanation, rather than indirectly via its effects on CM derived from CAFs as proposed. The direct effect of CR13452 on CM stimulated migration BT549 cells can be measured by including it in the invasion assay. If there is a significant direct effect of CR13452, can the authors dialyse the CM through a low MW cutoff and/or measure the levels of CR13452 in the CM via mass spec? Otherwise this experiment could be omitted without jeopardizing the overall MS.

We have decided to keep this experiment in. We have done the controls requested. We show that CR13452 activity is removed following the 10 kDa concentration step, which parenthetically was done for all harvested conditioned culture media before adding to Matrigel (new Figure 5G). We omitted to mention explicitly in the original presentation of the results that all CM are filtered. It was mentioned in the methods, however. We now explicitly state in the results and methods that all CM, including those treated with CR13452, are filtered before using.

7. For Fig. 7, is there any precedent for this experiment, e.g. have CAFs been co-injected with tumor cells in a tail vein injection metastatic colonization model? If so, this work should be cited. If not, this experiment raises some interesting questions that should be explored. Are the CAFs present in metastatic colonies? If not, where are they and how plausible is it that a relatively small number of CAFs (10e5) could exert paracrine effects systemically? Must the CAFs be co-injected into the tail vein or could they be implanted subcutaneously and exert the same effect on lung colonization of PyMT tumor cells?

© 2021. Published by The Company of Biologists under the terms of the Creative Commons Attribution License (https://creativecommons.org/licenses/by/4.0/).

We have done new experiments to address these questions (see revised Figure 7). We genetically marked WT CAFs with RFP and co-injected with tumor cells (tail vein IV). We then scored for the presence of exogenous CAFs (RFP+ cells) in lungs 2 days after injection and again at 7 days after injection, when lungs were scored for tumors. At day 2 CAFs are present and readily detectable. But by day 7 they are virtually gone. Our analysis cannot enumerate the total number of CAFs present in the lungs, however. So whether they proliferate in vivo or not is not known.

Minor concerns

MW markers on Fig. 1B. How were Western blot signals quantified?

Western blots were quantified via densitometry. This oversight has been corrected in the methods. We have added MW markers to panels where appropriate.

Address discrepancy in differences in levels of DDR2 detected in Fig. 1B and Fig. 1E between shSCR and shDDR2 + rescue constructs. Fig.1E looks much more than the 3-4 fold difference claimed for Fig. 1B. Does this suggest that overexpressed mutant DDR2 rescue constructs are trafficking to the surface much more efficiently than endogenous DDR2? What about WT DDR2 rescue in this assay?

Yes, the ΔKD mutant has a much higher level of rescue expression than either WT or K608E rescues. This has now been pointed out in the presentation of the results. We have not studied the trafficking of the various rescue isoforms.

This sentence (lines 141-42) is puzzling in light of the data presented in Fig. S1: "These analyses suggested that in all cell lines there was a quantitative difference in signaling, but no unique or qualitative signaling differences." The authors should clarify their interpretation of this data. Given the limits of this experiment, and that none of the observations from the array were validated further, this data might be removed as it does not advance the story.

This sentence has been removed. We have decided to leave the data in as it shows that K608E appears to signal to similar intermediates to WT, only quantitatively less. We did not detect any novel signaling intermediates activated or inhibited between WT and K608E cells .

For Fig. 6, the authors should explain in the methods how collective migration of tumor organoids is quantitated (perhaps seperately from other modes of migration?). This is not made apparent in the cited reference #3.

This has now been added to the revised methods. Nonetheless, we believe it was well-described in the Cancer Research 2019 paper.

For the discussion a couple of additional points could be considered: 1) Two alternatively spliced isoforms of DDR1 have been described (DDR1d and DDR1e) that are truncated or lack kinase activity. Has there been any effort to identify similar alternative spliceforms for DDR2? Does this also support kinase independent functions in the DDR family? 2) Any speculation on how DDR2 might be programming the secretome of CAFs?

To our knowledge there have not been any splice variants of DDR2 described. How DDR2 impacts CAF secretome production is the active area of ongoing and future work.

Reviewer 3:

Comments for the Author:
This reviewer does not request additional experimentation, just addressing the comments and some issues with data interpretation as well as expanding the discussion, if possible.

Specific points

1. Please can the authors add molecular weight markers to all the blot panels?

© 2021. Published by The Company of Biologists under the terms of the Creative Commons Attribution License (https://creativecommons.org/licenses/by/4.0/).

We have added MW markers to panels where appropriate.

2. Fig. 1A. Please indicate what are the different shapes intend to represent in the cartoon of DDR2 domains. Please also provide the last amino acid of DDR2 tagged with Myc. Please explain why was important to delete the entire KD also to address kinase function when compared to the point mutant kinase inactive.

The cytoplasmic tail truncation removes the entire tyrosine kinase domain, while retaining the intracellular juxta-membrane domain critical for receptor dimerization and collagen binding. The ΔKD mutant was chosen it will also be tyrosine kinase inactive but allows us to assess whether cytoplasmic signaling adapters recruited to this domain (in the absence of tyrosine phosphorylation) are important if there are tyrosine kinase independent actions of DDR2 (which we do observe). In full length construct myc is fused to aa E855. In ΔKD contrsct it is fused to G517. This has been added to the methods.

1B. Why is ERK1/2 shown, as a loading control? Seems odd considering could be regulated by collagen signaling, even total ERK. Please indicate in the legend that is a loading control. The legend states: "probed with the indicated antibodies" but none are indicated in 1B. Indicate the mass and what are the different species detected in that blot.

Cellular ERK1 or ERK2 levels do not change in response to collagen I induced activation of DDR2 (Nature Cell Biology, 2012). The activity of ERK2 (i.e., phosphorylation) is affected by DDR2 signaling, however, and required for DDR2 to stabilize Snail1. Labeling of Fig. 1B has been changed.

1D. Please label the DDR2 forms detected in the blot. Is unclear in this Figure whether collagen promotes oligomerization. Does it? If available, the authors could also show a blot of the parental cells with endogenous DDR2 under the same conditions, to demonstrate that their recombinant system reliably reflect the behavior of the natural endogenous receptor. Also, is unclear why such a long time (overnight) culturing on collagen was chosen as a method to stimulate receptor activation. Could this long time decrease total levels of receptors due to turnover/endocytosis? In other experiments a 6 h time of incubation was used.

In multiple collagen stimulation assays we have not observed significant differences between 6h and overnight stimulation times. It has been published (for DDR1) that collagen does indeed does induce preformed receptor dimer oligomerization and that this may be important for action. This has been stated and referenced in the results presentation section.

1E. Lacks the delta KD construct.

This has now been done and added to the figure.

2. The authors mentioned several breast cancer cell lines, but the data on expression and signaling focuses mostly on BT549. Is it the same in the other cell lines, even signaling? If no data are mentioned for the others (even as not shown), then please remove from the manuscript or precisely describe what cell lines were used in each experiment. If available, do the authors have information on DDR1 expression (or its potential induction by the culture on collagen, matrigel or expression of DDR2 constructs, or the conditioned media) in any of the cell lines used, including 4T1?

We have now done and shown parallel experiments with 4T1 breast tumor cells and mouse breast CAFs (secretome only). Results in BT549 and 4T1 cells are similar throughout. DDR1 is not expressed by BT549, HS578T, MDA-MB-231, and 4T1 cell lines. Trace amounts were detected in mouse CAFs.

3. Supp Fig 1. The phosphorylation array uses a different collagen concentration and status (gel) than the experiments used to establish the expression and activation of the constructs, can you provide an explanation why? Is it expected the system/constructs to behave in a similar fashion (extent of activation, surface amounts, multimerization, turnover) under 2D conditions? These data also lack no collagen, so could results represent collagen effects independent of DDR2? How significant are the differences observed? The delta KD and the K608E mutant appear to behave the

© 2021. Published by The Company of Biologists under the terms of the Creative Commons Attribution License (https://creativecommons.org/licenses/by/4.0/).

same (see point 2). quantitative difference in signaling, but no unique or qualitative signaling differences”. Regardless, this conclusion/data interpretation is unclear or written in a convoluted manner. Were there or were there no differences? Please be more specific/clear. But what does it mean?

Collagen-I gels were utilized for the intracellular signaling experiments due to the solubility of cell surface kinases bound to collagen. In collagen gel experiments, we observe an artificial decrease in DDR2 expression levels due to the insolubility of extracellular/receptor constructs. We have not observed any quantifiable difference between lower collagen concentrations and higher (gel) concentrations in extent of activation of DDR2 in cells. Surface modification experiments were performed on lower collagen concentrations due to the observed solubility issues. The referred conclusion sentence has been removed. We now simply describe the differences observed from the analysis.

4. Suppl. Fig. 2 and Fig 2A/B. Matrigel, which contains collagen IV, is used to assess invasion. Yet, DDR2 is a fibrillar collagen receptor and DDR2 supports invasion through this matrix. This is a very interesting part of the study because it may imply two different modes of action of DDR2 mediating invasion of basement membrane matrices and interstitial collagen matrices. Based on the data shown, the kinase mutant rescues invasion of Matrigel in the DDR2-silenced cells (2A) but not the truncated KD (2B). These data suggest that kinase activity is required for the pro-invasive action of DDR2 on Matrigel but a drastic truncation of the KD disrupts the kinase activity independent effect of the receptor on this matrix. While in the Discussion (lines 346-352) the authors discuss invasion of 3D collagen I, the Matrigel data are not discussed. Yet, it is nicely shown that conditioned medium from cells with reduced DDR2 inhibit invasion, suggesting that in this medium there is an inhibitor or lacks a stimulator of Matrigel invasion. Moreover, media from cells expressing the kinase defective DDR2 but not the wild type receptor recovers some of the invasive activity. However, the data interpretation of these interesting experiments in the Discussion is not well developed and hardly discusses how DDR2 can promote invasiveness of Matrigel in a kinase independent manner. Any idea/speculation what these media may contain based on the array data (Supp Fig 1)? Did the author test whether a DDR2 kinase inhibitor or neutralizing antibodies (if available) have an impact in these assays?

This is indeed an interesting observation and to us, not readily explainable. Yes there is no (very little) fibrillar collagen (DDR2 ligand) in Matrigels. However, matrigel is highly processed. Possibly DDR2 action in CAFs controls secretion and activity of extracellular proteases that could activate latent growth factor/cytokine in Matrigel. For example, it has been published that DDR2 affects the activity of MT1-MMP in cells, and we have shown that the action of DDR2 in ovarian cancer cells controls the protease processing of Fibronectin (ref 59). We have expanded our discussion around this.

Inhibition with CR13452 (novel DDR2 inhibitor - not another TKI, inhibits both kinase activity and other activities of DDR2) does block all DDR2 activity in all cells tested (Fig. 5E). As to how the secretome is working, this is the focus of our future work. We have performed multiple proteomic approaches and have identified promising candidates – both ECM proteins and cytokines. Many have been validated, but this will require much more work to fully understand and appreciate their mechanism(s) and biologic significance.

Why the conditioned media were obtained from cells cultured on collagen (line 449) and not on Matrigel too? The concentration of collagen used is not indicated also.

Cells were cultured on collagen I to activate DDR2. Collagen concentration was 1.5.mg/ml.

Can the authors speculate how kinase inactive DDR2 supports breast cancer invasion of a basement membrane-like matrix? Any ideas? Is it known whether breast cancer cells within the confines of a basement membrane (in situ carcinoma) express DDR2 in human tissues?

At present, we have no solid data supporting unique signaling by K608E vs WT. We do see more significant inhibition when the tyrosine kinase domain is removed, however. This might suggest that cytoplasmic signal adapter proteins recruited to the tyrosine kinase domain region that was removed play a role. But what these are we do not at present know. As to when DDR2 expression is activated in an invading tumor is an intriguing question. Our accumulated published

© 2021. Published by The Company of Biologists under the terms of the Creative Commons Attribution License (https://creativecommons.org/licenses/by/4.0/).

data to date support a model that DDR2 expression is induced in response to tumor cells acquiring an invasive program (e.g., EMT). We have shown in prior work that DDR2 is NOT required for EMT to take place, however. Rather, we believe that DDR2 is a mesenchymal gene turned on as tumor cells acquire more mesenchymal phenotypes. As such it sustains tumor cell invasion/migration through the fibrillar collagen-rich ECM. As discussed in comments to other reviewers perhaps DDR2 activity controls the secretion and activity of extracellular proteases that activate latent growth factors present in Matrigel (like TGFß for example). Published data for others have shown that DDR2 controls the activity of MT1-MMP in cells.

Could the findings in Fig 1 be different in terms of DDR2 regulation when cells are seed on Matrigel? Do the authors know how the DDR2 constructs behave (surface expression, oligomerization) when the same cells are seed on Matrigel-coated dishes, in similar experiments to those described in Fig. 1 (and Methods section line 435).

We did not perform these analyses in cells stimulated with Matrigel. As pointed out by the reviewer it is, perhaps, unusual that Matrigel would influence DDR2 activity (lack of fibrillar collagen, and collagen IV, that is present in Matrigel, is not a DDR2 ligand).

Are cells expressing the delta KD attaching more rapidly/strongly to collagen?

We have not assessed this but is an interesting question. That said our accumulated data, published and otherwise, is that DDR2 per se is NOT an adhesion receptor. That said, DDR2 does impact collagen-binding Integrin signaling (see Bayer et al., Elife, 2019) through inside-out regulation. So, we believe DDR2's major role is as a signaling receptor.

5. Fig. 4C can be interpreted that kinase activity contributes to the metastatic ability of the 4T1 cells because the KD mutant fails to restore full metastatic ability when compared to the shSCR or wild type DDR2 and there is no indication of the levels of the various DDR2 forms in the tumor cell in vivo. However, in spit that this is a great experiment, data interpretation appears a bit bias and could be improved by more precise language.

In new Fig. 4A we show that the level of rescue WT and K608E DDR2 in 4T1 cells used for in vivo experiments are roughly equivalent. In new experiments we have also assessed the ability of 4T1 cells depleted of DDR2 and rescued with ΔKD to support in vivo metastasis (see revised Fig. 4). They are equivalent to Ddr2-depleted control cells. By student t-test comparing K608E and ΔKD there are significantly less lung mets in the ΔKD implanted mice.

6. The authors argue their assay in Fig. 6 discriminate between single and collective migration. However, this is not explained in the results or method section. This should be described for the general reader.

This has been expanded in both the methods and results section. We show new data in Fig. 6E that tumor organoids migrate collectively. See also the image provided to Reviewer 1 that the organoids are migrating collectively.

7. Again, conditioned media seems to be a critical mediator of DDR2 action also in DDR2-manipulated CAFs. Can the authors discuss/speculate what could be the mechanism/factor?

This is the direction of ongoing future work. We have, through proteomic analysis, identified some candidates. Some are validated but much more work is required to dissect mechanism(s) and ascertain biologic significance. We find both ECM proteins and secreted cytokines whose secretion is controlled by the action of DDR2.

Minor points:

Line 109: "as determined by DDR2 immunoprecipitation total phosphotyrosine immunoblot" should read as determined by total DDR2 immunoprecipitation followed by immunoblot analyses for phosphotyrosine residues.

done

© 2021. Published by The Company of Biologists under the terms of the Creative Commons Attribution License (https://creativecommons.org/licenses/by/4.0/).

Line 348-9. The sentence: “Possibly cells expressing DDR2. DKD interact more avidly with collagen in part because they cannot be endocytosed” should replace by: because these truncated receptors cannot…. Otherwise, they refer to the cells.

done

Line 352: the dominant inhibitory manner the authors mention is unclear. What could the target be of the dominant effect? Apparently, there is no wild type DDR2. Perhaps this sentence (lines 351-352) should be followed by the reasoning described on line 353, without starting a new paragraph.

We have expanded our discussion of possible dominant inhibitor function of ΔKD in particular. Because all cell lines, except mouse CAFs, were generated using shRNA technology there is likely trace WT remaining (never 100% deletion). However, when we use genetic null CAFs (no DDR2 expression) rescued with mutants we do not see any dominant inhibitory activity. But this was only for secretome analyses.

Fig 2C and 2F could be supplemental.

Yes, but we have left them in Figure 2 for the time being. If space limitations, we are happy to move to supplemental data.

Line 144. The authors use migration/invasion as co-dependent mechanisms. While invasion may require cell migration, highly migratory cells may not necessarily be invasive. For instance, lack of a productive proteolytic system may compromise matrix penetration. Therefore, because the authors have not determined effects on migratory activity and their assay supposedly reflects invasion, I suggest removing the migration/invasion term.

We have tried to remove all invasion/migration terms, unless referring to both processes, in general. In the revised manuscript we now refer to movement through Matrigel as invasion and movement through ECM collagen I as migration.

<u>Second decision letter</u>

MS ID#: JOCES/2021/258431

MS TITLE: Tyrosine kinase independent actions of DDR2 in tumor cells and CAFs influence tumor invasion, migration, and metastasis

AUTHORS: Craig E Barcus, Priscilla Y Hwang, Vasilios Morikis, Audrey Brenot, Patrick Pence, Maria Clarke, and Gregory D. Longmore
ARTICLE TYPE: Research Article

We have now reached a decision on the above manuscript.

To see the reviewers' reports and a copy of this decision letter, please go to: https://submit-jcs.biologists.org and click on the 'Manuscripts with Decisions' queue in the Author Area. (Corresponding author only has access to reviews.)

As you will see, the reviewers gave favorable reports but one referee raised a single issue regarding interpretation of the statistical analysis in Figure 4C. Their comment is that the one-way ANOVA analysis (Tukey post hoc) does not indicate that lung metastasis is significantly different between the 4T1 cells rescued with the K608E mutant and the Î”KD mutant. But in the text there is reference to a t-test that does indicate significance. Please address this issue by clarifying and/or modifying the text appropriately.

© 2021. Published by The Company of Biologists under the terms of the Creative Commons Attribution License (https://creativecommons.org/licenses/by/4.0/).

We are aware that you may be experiencing disruption to the normal running of your lab that makes experimental revisions challenging. If it would be helpful, we encourage you to contact us to discuss your revision in greater detail. Please send us a point-by-point response indicating where you are able to address concerns raised (either experimentally or by changes to the text) and where you will not be able to do so within the normal timeframe of a revision. We will then provide further guidance. Please also note that we are happy to extend revision timeframes as necessary.

Please ensure that you clearly highlight all changes made in the revised manuscript. Please avoid using 'Tracked changes' in Word files as these are lost in PDF conversion.

I should be grateful if you would also provide a point-by-point response detailing how you have dealt with the points raised by the reviewers in the 'Response to Reviewers' box. Please attend to all of the reviewers' comments. If you do not agree with any of their criticisms or suggestions please explain clearly why this is so.

Reviewer 1

Advance summary and potential significance to field

In this manuscript, Barcus et al report an interesting kinase independent mechanism of action of the collagen receptor DDR2 in both tumor and stromal cells in breast cancer. The results of this manuscript are of special interest to the field of TKI therapeutics in cancer, as they might explain why treatments based on tyrosine kinase inhibitors fail to be efficient in breast cancer patients, and open the door to the designing of new therapies against non-kinase dependent functions of RTKs

Comments for the author

The authors have addressed most of my main concerns. I believe that the manuscript is now suitable for publication.

Reviewer 2

Advance summary and potential significance to field

In the revised manuscript Barcus et al. have substantially addressed prior concerns. The data supports the idea that tyrosine kinase-independent functions of DDR2 support breast cancer migration, invasion, and metastasis. Importantly conclusions that were previously based on one cell line have been extended to other breast cancer cell lines supporting the generality of the findings. The authors should be commended on making such a thorough response that clearly enhanced the impact of this work, advancing the field on the role of DDR2 in metastatic disease. This paper will serve as a point of departure to better define the non-tyrosine kinase functions of DDR2 that support metastasis. In particular the findings demonstrating the role of DDR2 in paracrine actions in tumor cells and CAFs will be a particularly interesting area of future study

Comments for the author

There is only one final comment about Figure 4C that the authors should address in some way. It appears that the one-way ANOVA analysis (Tukey post hoc) does not indicate that lung metastasis is significantly different between the 4T1 cells rescued with the K608E mutant and the ΔKD mutant. It is however mentioned in the text that: "Student t-test comparison of K608E and ΔKD cells indicated that ΔKD cells developed less lung metastases (Fig. 4C)." Strictly speaking, switching statistical methods like this to support a conclusion is not appropriate. If this is not an accurate read of the situation here, the authors should clarify.
Although the data look like there might be a trend toward a difference, and it is acknowledged that the one-way ANOVA is a more stringent test than a Student's t-test and so may obscure real differences, a textual change is recommended here. The authors should acknowledge that despite

© 2021. Published by The Company of Biologists under the terms of the Creative Commons Attribution License (https://creativecommons.org/licenses/by/4.0/).

the suggestion of a difference, the ANOVA does not show that the constructs are significantly different. This point might then come up in the discussion where it could be pointed out that since this experiment (4C) did not include CAFS it may have underestimated the role of the kinase-independent functions of DDR2 on metastasis in vivo.
The alternative of doing a repeated animal study with a much larger number of animals to tease out a small difference is not recommended here.

Reviewer 3

Advance summary and potential significance to field

This paper provides a new insight into the action of a unique collagen kinase receptor, DDR2, that are independent of its kinase activity in regulation of malignant properties. This is important considering the efforts of targeting kinase activity. As such the study will make a significant contribution to the field.

Comments for the author

The authors have properly addressed my comments.

Second revision

Author response to reviewers' comments

Response to Reviewers:
Reviewer 2 Comments for the author
There is only one final comment about Figure 4C that the authors should address in some way. It appears that the one-way ANOVA analysis (Tukey post hoc) does not indicate that lung metastasis is significantly different between the 4T1 cells rescued with the K608E mutant and the ΔKD mutant. It is however mentioned in the text that: "Student t-test comparison of K608E and ΔKD cells indicated that ΔKD cells developed less lung metastases (Fig. 4C)." Strictly speaking, switching statistical methods like this to support a conclusion is not appropriate. If this is not an accurate read of the situation here, the authors should clarify.
Although the data look like there might be a trend toward a difference, and it is acknowledged that the one-way ANOVA is a more stringent test than a Student's t- test and so may obscure real differences, a textual change is recommended here. The authors should acknowledge that despite the suggestion of a difference, the ANOVA does not show that the constructs are significantly different. This point might then come up in the discussion where it could be pointed out that since this experiment (4C) did not include CAFS it may have underestimated the role of the kinase-independent functions of DDR2 on metastasis in vivo.
The alternative of doing a repeated animal study with a much larger number of animals to tease out a small difference is not recommended here.
In response, we have now removed the text referring to the t-test comparison between K608E and ΔKD from the text. See new statement of result on the bottom of page 9 - highlighted.

Third decision letter

MS ID#: JOCES/2021/258431

MS TITLE: Tyrosine kinase independent actions of DDR2 in tumor cells and CAFs influence tumor invasion, migration, and metastasis

© 2021. Published by The Company of Biologists under the terms of the Creative Commons Attribution License (https://creativecommons.org/licenses/by/4.0/).

AUTHORS: Craig E Barcus, Priscilla Y Hwang, Vasilios Morikis, Audrey Brenot, Patrick Pence, Maria Clarke, and Gregory D. Longmore
ARTICLE TYPE: Research Article

I am happy to tell you that your manuscript has been accepted for publication in Journal of Cell Science, pending standard ethics checks.

© 2021. Published by The Company of Biologists under the terms of the Creative Commons Attribution License (https://creativecommons.org/licenses/by/4.0/).
